# Examining Quadratic Relationships Between Traits and Methods in Two Multitrait-Multimethod Models

**DOI:** 10.3389/fpsyg.2019.00353

**Published:** 2019-03-14

**Authors:** Fred A. Hintz, Christian Geiser, G. Leonard Burns, Mateu Servera

**Affiliations:** ^1^Department of Psychology, Utah State University, Logan, UT, United States; ^2^Department of Psychology, Washington State University, Pullman, WA, United States; ^3^Department of Psychology, University of the Balearic Islands, Palma, Spain

**Keywords:** structural equation modeling, multiple rater, multitrait-multimethod (MTMM) analysis, latent moderated structural equations, latent difference model, latent means model

## Abstract

Multitrait-multimethod (MTMM) analysis is one of the most frequently employed methods to examine the validity of psychological measures. Confirmatory factor analysis (CFA) is a commonly used analytic tool for examining MTMM data through the specification of trait and method latent variables. Most contemporary CFA-MTMM models either do not allow estimating correlations between the trait and method factors or they are restricted to linear trait-method relationships. There is no theoretical reason why trait and method relationships should always be linear, and quadratic relationships are frequently proposed in the social sciences. In this article, we present two approaches for examining quadratic relations between traits and methods through extended latent difference and latent means CFA-MTMM models (Pohl et al., 2008; Pohl and Steyer, 2010). An application of the new approaches to a multi-rater study of the nine inattention symptoms of attention-deficit/hyperactivity disorder in children (*N* = 752) and the results of a Monte Carlo study to test the applicability of the models under a variety of data conditions are described.

## Introduction

In psychology, researchers frequently examine the validity of tests and measurements they use. Convergent and discriminant validity are two aspects of validity that researchers typically study (American Educational Research Association et al., 2014). Evidence for convergent validity is provided when different measures (or “methods of measurement”) of the same psychological construct are strongly related (Cronbach and Meehl, 1955; Campbell and Fiske, 1959). Evidence for discriminant validity is provided when measures of different constructs (that pertain to the same or different methods) are sufficiently distinct from each other (Campbell and Fiske, 1959). For example, Cole et al. (1996) examined the extent to which children’s self-reports of their depression were concordant with the reports of their parents, their teachers, and their peers, thus testing convergent validity. In the same study, the authors also examined discriminant validity by looking at the extent to which the relations between the ratings of child depression, academic competence, and social competence were inflated due to the use of the same reporter type.

A common approach for examining convergent and discriminant validity is called the multitrait-multimethod (MTMM) design (Campbell and Fiske, 1959). In an MTMM study, researchers gather data on multiple traits (e.g., depression, self-esteem, competence) that are assessed with multiple methods (e.g., self, parent, and teacher reports). According to Campbell and Fiske (1959), each variable represents a trait-method unit (TMU), as it reflects both trait and method (e.g., self-report of depression, parent report of self-esteem, etc.).

Convergent and discriminant validity are evaluated based on the correlation matrix that results from an MTMM design using specific criteria. Specifically, convergent validity is supported by strong correlations between ratings of the same trait by different methods. In contrast, discriminant validity is supported by correlations between different traits measured by the same method that are not too large.

The original correlation-based MTMM analyses has a number of limitations (Widaman, 1985). These limitations include a lack of correction for measurement error (unreliability) in the variables representing each TMU and the inability to relate trait and method effects to one another and to external variables. Confirmatory factor analysis (CFA) models are now widely used to analyze MTMM data, as they allow addressing many of the limitations of the original MTMM correlation matrix approach (Kenny, 1976; Browne, 1985; Widaman, 1985; Marsh and Hocevar, 1988; Eid, 2000). CFA uses latent variables to represent trait and method effects and corrects for random measurement error. Furthermore, CFA explicitly expresses trait and method effects in terms of latent factors, thus allowing researchers to study relationships between trait, method, and external variables.

A number of different CFA-MTMM models have been proposed in the literature. For example, the correlated-traits correlated-methods (CTCM) model (Jöreskog, 1971) includes as many trait and method factors as there are traits and methods in the study. The CTCM model allows correlations between the trait factors as well as correlations between the method factors, but no correlations between the trait and method factors (Widaman, 1985). The correlated-traits-uncorrelated-methods model (CTUM) has the same basic structure as the CTCM model, but assumes uncorrelated method factors (Marsh and Hocevar, 1983). The correlated-traits-correlated-uniqueness (CTCU; Kenny, 1976) model includes as many trait factors as there are traits in the study. Instead of including method factors, the CTCU model allows correlations between the measurement error variables that pertain to the same method.

Most CFA-MTMM models posit that trait factors can be correlated with other trait factors, and that method factors can be correlated with other method factors. However, correlations between trait and method factors are typically restricted to zero, either for ease of interpretation, statistical reasons, or based on the definition of method factors as regression residuals (e.g., Eid, 2000). Nonetheless, such trait-method correlations could be present and meaningful in practical applications of the MTMM approach. Specifically, trait-method correlations indicate that method effects are larger or smaller depending on the level of the trait.

An example of a potential trait-method relationship is that method effects pertaining to peer reports of children’s extraversion could be related to the children’s level of extraversion. That is, when an individual scores low in extraversion, it may be more difficult for his or her peers to judge the extent to which they prefer to be alone rather than with others, and the discrepancy between peer and self-report may be higher.

In contrast, when an individual is high in extraversion, it may be easier for his or her peers to see that an individual prefers to be around others and the peer and self-reports might show greater agreement (higher convergent validity). Method effects (discrepancies between peer and self-reports) would therefore be larger at lower levels of the trait and smaller at high levels of the trait. This would be reflected in a negative relationship between the trait (extraversion) and the method effect (peer report vs. self-report): the higher the trait score, the smaller the method effect.

Effects like these are ignored in the most frequently used MTMM models (Podsakoff et al., 2003). In most MTMM models, it is implicitly assumed that trait levels are unrelated to method effects. This is either because the method effects are defined in such a way that they must be uncorrelated with the trait factors (e.g., Eid, 2000), or because of concerns with model identification, convergence, overfitting, or interpretability of model parameters (Widaman, 1985; Marsh and Grayson, 1995). In other words, these restrictions are typically chosen for statistical expediency rather than for substantive reasons. As noted by Marsh and Grayson (1995), trait-method correlations are typically constrained to zero in CFA-MTMM models “to avoid technical estimation problems and to facilitate decomposition of variance into trait and method effects, not because of substantive likelihood or empirical reasonableness” (p. 181). In fact, the creators of the MTMM approach themselves noted in a later paper that “method and trait or content are highly interactive and interdependent” (Fiske and Campbell, 1992). In summary, it is plausible that trait and method factors could be correlated, yet most commonly used statistical models for MTMM data do not allow the estimation of correlations between trait and method factors.

Early applications of CFA-MTMM models found large trait-method correlations when using the CTCM approach (Boruch and Wolins, 1970; Kalleberg and Kluegel, 1975; Schmitt, 1978). However, the CTCM approach is prone to both conceptual as well as convergence, admissibility, and interpretation problems (Marsh, 1989; Geiser et al., 2008). The addition of correlations between trait and method factors compounded these problems (Marsh, 1989; Kenny and Kashy, 1992). Other CFA-MTMM approaches have been developed to avoid the problems typically encountered with the CTCM model (Eid, 2000; Pohl et al., 2008; Pohl and Steyer, 2010).

Recently, two CFA-MTMM models have been proposed that allow for the estimation of trait-method correlations and that do not show the same estimation and identification problems as the CTCM approach: the latent difference (LD) and the latent means (LM) model (Pohl et al., 2008; Pohl and Steyer, 2010). These models are based on classical test (true score) theory and can be used to estimate linear relationships between trait and method factors. Linear relationships between traits and methods indicate that method effects increase or decrease as a function of the trait level. One shortcoming of the LD and LM models in their current form is that quadratic relationships between traits and methods cannot be tested. A quadratic relationship could exist, for example, if parent and teacher reports of children’s attention-deficit/hyperactivity disorder (ADHD) symptoms are less discrepant at low and high symptom levels, but more discrepant at intermediate symptom levels. This may be the case because certain ADHD symptoms may not be visible to the parents, but may be visible to the teachers. In contrast, children with very low or very high levels of ADHD may be correctly identified by both parents and teachers. In general, method effects may be weaker at lower or higher levels of a trait as individuals with “extreme” trait levels or symptoms may show stronger convergence of, for example, external observers due to the greater visibility of their symptoms compared to individuals with more moderate trait levels.

Currently, such quadratic relationships cannot be tested within the CFA-MTMM framework. Some methodologists have previously noted that theoretical models involving quadratic effects are frequently posited in psychology, but that a methodological understanding of how to test those models is lacking (Aiken and West, 1991). Ignoring quadratic trait-method relationships could result in either disregarding a true threat to convergent validity, or inappropriately concluding that convergent validity is lower than it truly is. In this paper, we propose an approach for examining quadratic trait-method relations with MTMM data.

The remainder of the paper is organized as follows: we first review a basic CFA-MTMM model for multiple-indicator data that served as the basis for defining the LD and LM models. Second, we present the standard LD and LM models and compare them in terms of their ability to represent linear trait-method relationships. Third, we describe extensions of the LD and LM models that allow analyzing quadratic relationships between traits and methods. Fourth, an application of the linear and quadratic LD and LM models to real data is presented. Lastly, we briefly discuss the results of a Monte Carlo simulation study examining the performance of the model extensions under a larger set of conditions.

## The Basic Tmu, Ld, and Lm Models

In this section, the mathematical definitions and assumptions characterizing the LD and LM models are presented. To simplify our presentation, we consider a design in which just one trait is measured by just two methods, as this is the simplest possible way to examine convergent validity (Geiser et al., 2012) as well as trait-method relationships. The extension to additional traits and methods is straightforward. In addition, we consider models with multiple indicators in each TMU in line with Marsh and Hocevar (1988). This approach is preferable to single-indicator designs, because it allows for the examination of trait-specific method effects and latent (instead of observed) variables representing each TMU (Marsh and Hocevar, 1988). Before introducing the LD and LM models, we discuss the basic TMU model that serves as the basis for formulating both the LD and LM models.

### The Basic TMU Model

**FIGURE 1:**
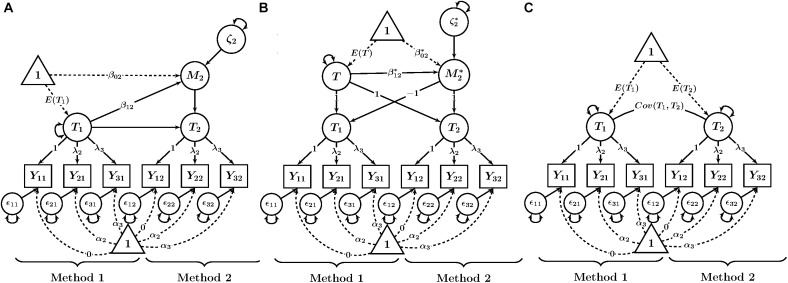
Path diagrams of CFA-MTMM models for examining linear trait-method relationships. **(A)** Basic TMU model. **(B)** Latent difference (LD) model. **(C)** Latent means (LM) model. *Y_im_* = observed variable (*i* = indicator, *m* = method); λ_i_ = factor loading; α_i_ = intercept; 𝜀_im_ = measurement error variable. T_m_ = trait as measured by method *m*; *M*_2_ = method factor for Method 2 in the LD model; *T* = common trait factor in the LM model; $M_{2}^{*}$ = method factor in the LM model; β_0m_, ${\text{β}}_{0m}^{*}$ = latent regression intercept coefficient; β_1m_, ${\text{β}}_{1m}^{*}$ = latent regression slope coefficient; ζ_2_, $\zeta_{2}^{*}$ = latent residual variables.

Each method in our example uses three observed variables (indicators) *Y_im_*, where *i* indicates the observed variable and *m* indicates the method (e.g., self-report) and *m* = 1, …, *K*. Note that no index for the trait is required here, given that for simplicity, we consider only one trait. In the basic TMU model (see Figure 1A for a path diagram), all variables *Y_im_* that share the same method *m* measure a common method-specific latent factor (true score variable) *T_m_* as well as a measurement error variable *𝜀_im_*. The measurement equation for each variable is in line with a congeneric measurement model and is given by:

(1) $$Y_{im}=\alpha_{im}+\lambda_{im}T_{m}+{\text{ε}}_{im}$$

where α*_im_* is a constant intercept parameter and λ*_im_* is a constant factor loading parameter. The *T_m_* factor can be interpreted as a common true score variable in the sense of classical test theory. It represents true score variance common to all indicators measuring the same trait with method *m*.

For model identification purposes, researchers often fix the intercept of one indicator per TMU to zero and the factor loading of the same indicator to unity to identify the scale and mean of each *T_m_* factor. We follow this approach in the present paper. The means, variances, and covariances of the *T_m_* factors can then be freely estimated. The *𝜀_im_* variables have variances estimated, but have means of zero by definition as measurement error variables (Steyer, 1989). Furthermore, all error variables *𝜀_im_* are uncorrelated with all other error variables as well as with all *T_m_* factors.

In the basic TMU model, all *T_m_* factors are allowed to correlate. High correlations of *T_m_* factors for the same trait but different methods indicate strong convergent validity across methods. In contrast, weak correlations indicate the presence of substantial method effects (e.g., disagreement between reporters). One advantage of the basic TMU model over classical MTMM analysis with observed correlations is that the *T_m_* factor correlations represent latent correlations that have been corrected for measurement error and are thus more accurate.

Another advantage of the basic TMU model is that is allows researchers to test for measurement equivalence of the loadings and intercepts of the observed indicators across methods (also referred to as *strong factorial invariance*; Widaman and Reise, 1997; Cheung and Rensvold, 2002). Equal loadings and intercepts are required for meaningfully comparing *T_m_* factor means and variances across methods (Geiser et al., 2014). Non-equivalent intercepts and/or loadings across methods would mean that differences in, for example, latent variable means across methods may be due to differences in the measurement structure (e.g., differences in item difficulty between methods) rather than true score mean differences (Pohl et al., 2008; Pohl and Steyer, 2010; Geiser et al., 2014).

Prior to fitting either the LD or LM models (which use LD scores), invariance of the intercepts and loadings should be tested in the basic TMU model. Because strong measurement equivalence is a prerequisite for a meaningful interpretation of LD scores. In our subsequent presentation, we assume that strong measurement equivalence has been established. Figure 1 thus refers to the α*_im_* and λ*_im_* parameters with only a subscript *i* (i.e., we assume the intercepts and loadings to be invariant across methods). After establishing measurement equivalence in the TMU model, researchers can move on to examine method effects in the more complex LD and LM models.

One limitation of the basic TMU model is that it does not contain latent variables representing method effects (i.e., there are no method factors). Therefore, method effects cannot be examined directly in terms of latent factors and cannot be related to external variables. The LD and LM models are equivalent to the basic TMU model, but address this limitation by including method factors. Furthermore, the basic TMU model uses “method-specific” (rather than “common”) trait factors (i.e., the *T_m_* factors do not reflect “pure” trait effects, but contain method-specific effects as well). The LM model also addresses this second limitation.

### The LD Model

The LD model defines a method factor as the difference between a *T_m_* variable and another *T_m_* variable that is defined to serve as reference (Pohl et al., 2008; Geiser et al., 2012). Without loss of generality, we choose the first method (*m* = 1) to serve as reference so that *T*_1_ denotes the reference true score variable. Without making any restrictive assumptions, we can decompose each non-reference *T_m_* variable as follows:

$$T_{m}=T_{1}+(T_{m}-T_{1}).$$

The LD score variable (*T_m_* – *T*_1_) is defined to be the method factor for comparing Method *m* to the reference method for a given trait:

$$M_{m}\equiv (T_{m}-T_{1}).$$

The LD approach thus requires the selection of a reference method against which another method is contrasted. It has been suggested that in MTMM models requiring the selection of a reference method a “gold standard” method or a method with a clear structural difference from the other methods should be chosen as the reference method (Geiser et al., 2008). For example, when self- and other-reports are used in a study, it would be natural to select self-report as the reference method unless there are theoretical or practical reasons to prefer a different method (e.g., in studies of very young children, parent reports might be seen as more dependable than the children’s self-reports).

The mean and covariance structure of the trait and method variables in the LD model are unrestricted, such that the means, variances, and covariances can be estimated for all trait and method factors. Individual scores on the method factor *M_m_* in the LD model indicate the difference between the non-reference method and the reference method for a given individual. For example, if both mothers and fathers rate a child’s inattention level with mother report used as the reference method, and the scores of the latent TMU variables are 1 for the mother report and 1.5 for the father report, then the value of the method variable for that child is 1.5–1 = 0.5. This would indicate that the child’s score is overestimated by 0.5 by his or her father relative to the mother’s report.

The mean of the method factor *E*(*M_m_*) indicates the average difference between the non-reference method as compared to the reference method. For example, if the mean of the method factor is 0.5, then, on average, fathers in the sample rate children 0.5 points higher than all mothers in the sample. The variance of the method factor *Var*(*M_m_*) indicates the spread of the individual method effect scores. For example, if the variance of the method factor is 0.81, then the standard deviation is $\sqrt{0.81}$ = 0.9, which means that each discrepancy is, on average, 0.9 points away from the mean difference score. Therefore, even though fathers rate 0.5 points higher than mothers on average, it is likely that if the mother rates the child as a 1, a substantial number of father ratings for that child (68%) fall anywhere between 0.6 and 2.4.

High convergent validity would be supported if the mean of the method factor was close to zero and the variance of the method factor was relatively small. This would indicate that the scores from both methods tend to show strong agreement.

The covariance between the trait and method factors can be represented either as a covariance parameter or, equivalently, as a linear regression of the method factor on the trait factor, as follows:

$$M_{m}={\text{β}}_{0m}+{\text{β}}_{1m}T_{1}+{\text{ζ}}_{m},$$

where β_0_*_m_* is a constant intercept parameter, β_1_*_m_* is a constant regression slope parameter, and *ζ_m_* represents a regression residual variable with a mean of zero that reflects variance in *M_m_* that is not accounted for by *T*_1_. The β_1_*_m_* parameter represents the strength of the linear relationship between the trait factor and the method factor. Figure 1B shows a path diagram of this model with the mean structure included.

When the LD trait-method relationship is parameterized as a linear regression, the mean and variance of the method factor and the covariance of the method factor with the trait factor are represented by the intercept β_0_*_m_*, the regression slope β_1_*_m_*, and the latent residual variance *Var*(*ζ_m_*). The intercept β_0_*_m_* reflects the expected value of the method factor when the reference-method trait level is zero. The regression slope β_1_*_m_* indicates the direction and degree of a potential linear relationship between a method factor and the reference trait factor.

For example, a positive regression slope β_1_*_m_* would show that at higher levels of inattention as rated by mothers (reference method), fathers tend to overestimate inattention more than for lower levels of inattention. A negative regression slope β_1_*_m_* indicates that, as levels of the trait as measured by the reference method increase, the method scores become smaller. That is, when mothers rate children’s inattention as high, fathers may tend to either overestimate inattention less strongly (difference scores get closer to zero), or underestimate inattention more strongly (difference scores become more negative).

The completely standardized version of the slope β_1_*_m_* is equal to the correlation between *T*_1_ and *M_m_* and can be used as an effect size measure for the strength of the linear association between trait and method factors. The latent residual variance *Var*(*ζ_m_*) reflects the variability in the method factor that is not accounted for by the linear relationship with the reference-method trait factor.

### The LM Model

An alternative to the LD model is the LM model (see Figure 1C), which does not require the selection of a reference method. In the LM model, a common trait factor is defined as the mean of the trait factors across all methods. Method factors are defined as deviations from the mean (Pohl and Steyer, 2010). The LM model may therefore be more meaningful when a clear reference method is not available, or the methods are not clearly distinguishable (e.g., interchangeable judges), since the common trait factor is defined as the average of scores from both methods.

Formally, the trait variable for a LM model in which one trait is measured by two methods is defined as:

$$T\equiv \frac{T_{1}+T_{2}}{2},$$

where *T* is the common trait factor. The method effect variables are defined as:

$$\begin{array}{l} M_{1}^{*}\equiv T_{1}-T\text{ and}\\ M_{2}^{*}\equiv T_{2}-T, \end{array}$$

where we denote the method factors in the LM model as $M_{m}^{*}$ to differentiate them from the method factors in the LD model, which are defined as differences from a reference method rather than differences from an overall average. Simple algebraic manipulation yields the structural equations for the TMU factors:

$$\begin{array}{l} T_{1}=T+M_{1}^{*}\\ T_{2}=T+M_{2}^{*}. \end{array}$$

Given that *T* is defined as the mean of the trait variables across methods, the deviations from the trait factor add up to 0 by definition. In our design with just two methods, we have

$$M_{1}^{*}+M_{2}^{*}=0$$

Therefore, the two method variables are equal in magnitude but oppositely signed^1^ such that

$$M_{1}^{*}=-M_{2}^{*}.$$

Given the deterministic relationship between method factors, only *K* – 1 method factors need to be included in the analysis. For example, in our design with two methods, we need only one method factor. Without loss of generality, we replace $M_{1}^{*}$ (i.e., the method factor for the first method) by -$M_{2}^{*}$ in the structural equation for the first TMU variable given above. Then, the structural model for the TMU factors can be re-written as:

$$\begin{array}{l} T_{1}=T-M_{2}^{*}\\ T_{2}=T+M_{2}^{*} \end{array}$$

It can be seen that the first TMU variable has an implicit loading of -1 on the method factor $M_{2}^{*}$, whereas the second TMU variable has an implicit loading of +1 on the method factor $M_{2}^{*}$. Both TMU factors have an implicit loading of +1 on the common trait factor *T*. This is illustrated in the path diagram in Figure 1C.

In the LM model, both *T*_1_ and *T*_2_ are completely determined by the *T* and *M* factors. Similarly to the LD model, the means, variances, and covariances of all latent variables are freely estimated. The covariance between the trait and method factors can also be parameterized as a linear regression of the method factor on the trait factor. In general, we have:

$$M_{m}^{*}={\text{β}}_{0m}^{*}+{\text{β}}_{1m}^{*}T+{\text{ζ}}_{m}^{*},$$

where ${\text{β}}_{0m}^{*}$ is a constant intercept parameter, ${\text{β}}_{1m}^{*}$ is a constant slope parameter, and $\zeta_{m}^{*}$ is a latent residual variable representing variability in $M_{m}^{*}$ that is not accounted for by the common trait factor *T*. Figure 1C shows a path diagram of the LM model with the mean structure and latent regression included.

In our case with just two methods, the mean of the method factor $E(M_{2}^{*})$ indicates the average deviation from the common trait for the method that has positive loadings on the $M_{2}^{*}$ factor. The individual scores on the method factor indicate ½ times the difference between each method’s scores for that individual. For example, if a mother reports a score of 2 for her child’s inattention and a father provides a rating of 3 for the same child, the score for that individual on the $M_{2}^{*}$ factor would be 0.5. The mean E($M_{2}^{*}$) of the method factor is the mean of these individual scores, and therefore the mean difference between the two methods is two times the mean of the $M_{2}^{*}$ factor.

The variance of the method factor *Var*($M_{2}^{*}$) indicates the spread of the method factor scores. Since the $M_{2}^{*}$ factor represents half the distance between the TMU scores, the variance of $M_{2}^{*}$ is ddd the size of the variance of the method factor in the LD model. For this reason, it may be easier to interpret the standard deviations of the $M_{2}^{*}$ factor instead of the variance. The standard deviation of the $M_{2}^{*}$ indicates how far away the typical individual $M_{2}^{*}$ score is from the mean of $M_{2}^{*}$, and can be compared to the standard deviation of the *T* variable to judge the size of the effect. Convergent validity is supported by a relatively low mean and variance (or standard deviation) of $M_{2}^{*}$.

A potential linear relationship between the $M_{2}^{*}$ factor and the common trait factor *T* can be examined by analyzing the ${\text{β}}_{0m}^{*}$, ${\text{β}}_{1m}^{*}$, and V ar($\zeta_{m}^{*}$) parameters. The intercept parameter ${\text{β}}_{0m}^{*}$ indicates the expected value of the $M_{2}^{*}$ variable when the common trait factor is 0. The regression slope parameter ${\text{β}}_{1m}^{*}$ represents the direction and magnitude of the linear relationship between the common trait *T* and the method variable $M_{2}^{*}$. Higher values of ${\text{β}}_{1m}^{*}$ indicate that values of the $M_{2}^{*}$ factor increase more steeply as values of the trait increase. The completely standardized version of ${\text{β}}_{1m}^{*}$ gives the correlation between *T* and $M_{2}^{*}$. The variance of the residual variable V ar($\zeta_{m}^{*}$) indicates how much of the variance in the $M_{2}^{*}$ variable is linearly unrelated to the trait variable.

### Comparison of the LD and LM Models for Analyzing Trait-Method Relations

The LD and LM models are equivalent models that imply the same covariance and mean structure for a given set of data. The models are similar in that they both contain *K* – 1 method factors that are defined in terms of LD score variables. Both models allow trait and method factors to be correlated. In both models, the covariances between the trait and method factors can be modeled as a linear regression of the method factors on the reference trait factor.

The trait and method factors, however, are defined differently in each model, which means that the trait-method relationship has a different meaning across models. Although model fit is identical for the linear LD and LM models for a given set of data, the means and variances of the trait and method factors as well as the size and direction of the trait-method relationship (covariance) can be very different in each model (In Appendix A, we provide more formal details on how each of the structural parameters of the LD and LM models can be derived from the baseline TMU model).

In the LD model, the trait factor is defined to be the trait as measured by a reference method, and the method factor is the difference between the reference method TMU and the non-reference method TMU. Therefore, the trait-method relationship is the relationship between the level of the reference method TMU and the difference between the two TMU variables. Consider a mother and father rating a child’s level of inattention on a five-point scale, where 1 indicates lowest and 5 indicates the highest level of inattention symptoms. Assume that after correcting for measurement error, the mother rating of inattention is 2.5, and the father rating is 1.5. If we were to use the LD model for this data and select the mother report as the reference method, the trait factor score for that child would be 2.5, and the method factor score for the father deviation from the mother report is 1.5–2.5 = –1, indicating that the true father report underestimates the child’s inattention by 1 point relative to the true mother report.

In general, a positive linear relationship between the reference TMU factor and the method factor in the LD model indicates that as the scores on the reference factor increase, there is a tendency for the method factor scores to increase also. For example, for higher levels of inattention as rated by mothers, the difference between father and mother scores may tend to increase. In contrast, a negative linear relationship between trait and method factor would indicate that as inattention scores go up, the difference scores between father and mother ratings become smaller.

Note that method factor scores can take on positive as well as negative values (or the value of zero when there is no discrepancy between reporters). Therefore, “smaller” here could also mean “more strongly negative.” That is, rather than considering the absolute value of the difference, the model deals with raw difference scores. A small value of the difference score therefore does not necessarily indicate a small discrepancy between reporters. Such a value (i.e., a strongly negative method factor score) could in fact indicate a strong discrepancy between reporters. For this reason, it is important to consider the range of scores, for example, by using scatter plots of estimated factor scores as we show later in our illustrative application.

In the LM model, the trait factor is defined as the mean of the true score variables across methods for the same trait, and the method factors ($M_{m}^{*}$) are defined as the deviations of a specific true score variable from the mean true score (trait) variable. In the LM model, the trait factor therefore has a completely different meaning than in the LD model, as it represents the average across all true score variables pertaining to the same trait. In the two-method case, $M_{2}^{*}$, however, has a similar meaning to *M* in the LD model, as $M_{2}^{*}$ represents ½ of the difference between the two TMUs.

If we consider the above example using the LM model instead of the LD model, a true mother rating of 2.5 and a true father rating of 1.5 would result in a trait factor score of 2 as well as method factor scores of +0.5 for the mother rating and -0.5 for the father rating. A positive correlation between the common trait factor and a method factor in the LM model indicates that as the value of the mean of mother and father ratings of inattention increases, the deviation of a specific reporter (e.g., fathers) from that average tends to increase also.

## Quadratic Relationships Between Traits and Methods

It is plausible that, in addition to linear relationships, quadratic relationships between traits and methods exist in practice. For example, at low and high levels of a trait, discrepancies between methods may be smaller than in the center of the trait distribution. Although plausible in many applications, to our knowledge, quadratic trait-method relations have not yet been tested and models have not yet been proposed to examine them. We now present extensions to the LD and LM models that allow for an examination of quadratic trait-method relationships.

### The LD Model With Quadratic Trait-Method Relationships

In order to examine quadratic relationships between trait and method factors, a squared term $T_{1}^{2}$ with an additional regression slope coefficient β_2m_ is added to the structural regression equation in the LD model:

$$M_{m}={\text{β}}_{0m}+{\text{β}}_{1m}T_{1}+{\text{β}}_{2m}T_{1}^{2}+{\text{ζ}}_{m}.$$

**FIGURE 2:**
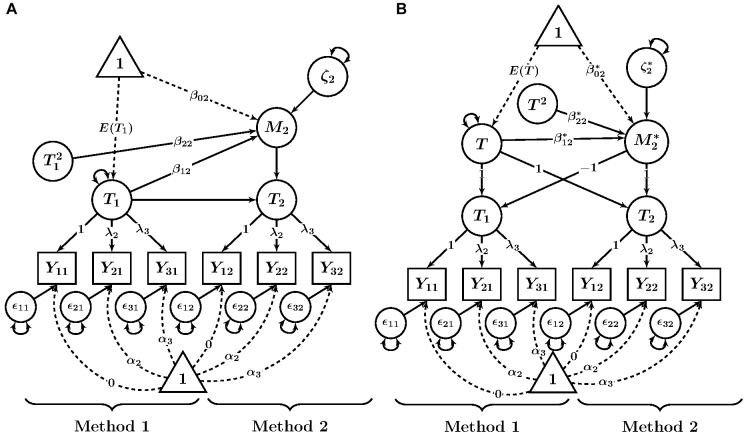
Path diagrams of quadratic extensions of CFA-MTMM models. **(A)** LD model. **(B)** LM model. *Y_im_* = observed variable (*i* = indicator, *m* = method); fff = factor loading; ggg = intercept; 𝜀_im_ = measurement error variable. *T_m_* = trait as measured by method *m*; *M*_2_ = method factor for Method 2 in the LD model; *T* = common trait factor in the LM model; $M_{2}^{*}$ = method factor in the LM model; β_0m_, ${\text{β}}_{0m}^{*}$ = latent regression intercept coefficient; β_1m_, iii, ${\text{β}}_{1m}^{*}$, hhh = latent regression slope coefficients; ζ_2_, $\zeta_{2}^{*}$ = latent residual variables.

Figure 2A shows a path diagram of this model. For β_2m_≠0, a quadratic relationship is present in the data. A value of β_2m_ > 0 indicates a u-shaped curve, whereas a value of β_2m_ < 0 indicates an inverted-u-shaped curve.

To determine the points at which the values of the method factor are implied to be zero and the maximum level of discrepancy, researchers should calculate the roots and the inflection point of the quadratic function. Note that they are only substantively meaningful if they are within the observed range of the measures. The inflection point is the value of the trait level at which the relationship between the two variables changes direction, and thus reflects the trait level that corresponds to the minimum or maximum level of the method effect. The formula for the inflection point is given by

$$\frac{-{\text{β}}_{1m}}{2{\text{β}}_{2m}}.$$

For example, for the quadratic function 0.6 - 0.7T_1_ + $0.1T_{1}^{2}+\zeta_{m}$, the inflection point is $\frac{0.7}{0.2}=3.5$. This means that below 3.5, values of the method factor decrease as the value of the trait factor increases. Above 3.5, values of the method factor increases as the value of the trait factor increase. The model-predicted minimum or maximum value of the method factor can be obtained by plugging the trait-factor value into the model equation. For the previous example, the predicted value of *M_m_* is thus 0.6 - 0.7 ⋅ (-3.5) + 0.1 ⋅ (-3.5)^2^ = -0.625. This means that for the previously given model-estimated formula, the maximum predicted value of *M_m_* is -0.625 and occurs when *T*_1_ takes on the value of 3.5. The size of this maximum predicted value should be interpreted in the units of the measure being examined.

The values of *T*_1_ for which the predicted value of *M_m_* is zero are called the roots of the function. The roots are given by:

$$\frac{-{\text{β}}_{1m}\pm \sqrt{{\text{β}}_{1m}^{2}-4{\text{β}}_{2m}{\text{β}}_{0m}}}{2{\text{β}}_{2m}}$$

There may be zero, one, or two roots. The roots of the method factor regression are only of substantive interest if they are within the range of the observed data. In the example given, the roots are *T*_1_ = 1 and *T*_1_ = 6. This means that method effects are predicted to be lowest at those levels of the trait.

In addition, a good way to interpret the quadratic function is to plot the trend in the range of the observed data and compare it with the linear model. In this way, the substantive significance (or lack thereof) of the quadratic trend will become more easily apparent.

### The Latent Means Model With Quadratic Trait-Method Relationships

The proposed LM structural model with a quadratic trait-method relationship is analogous to the previously described extension of the LD model and is given by:

$$M_{m}^{*}={\text{β}}_{0m}^{*}+{\text{β}}_{1m}^{*}T+{\text{β}}_{2m}^{*}T^{2}{\text{+ζ}}_{m}^{*}.$$

Figure 2B shows a path diagram of the quadratic LM model. The inflection point and roots of this function can be also be calculated in the same way as shown above for the LD model.

### Comparison of Quadratic Trait-Method Relationships in the LD and LM Models

It is well-known that the distribution of quadratic and interaction terms is not a normal distribution (Moosbrugger et al., 1997). In latent variable models with quadratic structural relationships, the endogenous latent variables and their indicators are therefore implied to be non-normally distributed (Klein and Moosbrugger, 2000). An important difference between the linear and quadratic LD and LM models is therefore that when the quadratic extension is added to the LD and LM models, it is no longer true that model fit will be the same for both types of models as we explain below.

The implied distribution of the TMU variables is multivariate normal for the linear LD and LM models. For the quadratic LD model, the observed variables for the reference TMU variable *T*_1_ are implied to be multivariate normal, and the observed variables for the non-reference TMU variable *T_m_* are implied to be non-normal. For the quadratic LM model, all observed variables for both TMU variables are implied to be non-normal. This is because the latent $M_{m}^{*}$ variable is implied to have a non-normal distribution, and both latent TMU variables are dependent on the $M_{2}^{*}$ variable. In this way, each model has a different implication for the distribution of the observed variables.

We recommend that researchers should not primarily rely on model fit indices when making a decision about whether to choose the quadratic LD or LM approach. Instead, the choice should be based on whether it makes more sense theoretically or substantively to contrast *K* – 1 methods against a reference (as in the LD model) or compare the methods to an overall average. When there are clear structural differences between methods and/or when one method differs from the remaining methods (e.g., self- versus other reports of depression; objective test score versus subjective ratings of intelligence), the LD model is typically the better choice, because an overall average is typically less meaningful in this case. When there is not a single clearly outstanding method or no clear difference between methods (e.g., multiple friend reports of depression; multiple test batteries to measure intelligence), the LM approach may be more useful.

### Estimation of Quadratic Relationships in the LD and LM Models

A variety of estimation methods have been developed to analyze quadratic relationships in structural equation models (for an overview, see Harring et al., 2012). In the present paper, we focus on the latent moderated structural equations (LMS) approach, which uses numerical integration to approximate the probability density of the quadratic term, and models the probability density of the dependent variables as a mixture of the normal and non-normal distributions in the independent variable part of the model (Klein and Moosbrugger, 2000). The expectation-maximation (EM) algorithm is then used to estimate the mixing proportions of the normal and non-normal densities in the dependent variable portion of the model (Klein and Moosbrugger, 2000). The technique has been shown to be unbiased and efficient compared with other techniques and is robust to moderate non-normality, although other estimation methods may be more robust to non-normality (Harring et al., 2012). Unlike some other methods, the LMS method does not provide an absolute measure of model fit, but likelihood ratio tests can be used to perform nested model tests against linear models without a quadratic term (Klein and Moosbrugger, 2000; Kelava et al., 2011). In addition, the LMS technique is readily available in the software package M*plus* (Muthén and Muthén, 1998-2015).

## Application

We now present an application of the quadratic LM and LD models to an actual empirical data set containing multi-rater reports of children’s ADHD inattention symptoms to demonstrate the estimation and interpretation of non-linear trait-method relationships. The Mplus syntax for all models is available from Appendix C.

### Sample

The data for this application come from a longitudinal study of childhood symptoms of ADHD (Burns et al., 2014). The sample consisted of children from 30 schools in Madrid and the Balearic Islands in Spain. Mothers, fathers, and teachers of the children rated the ADHD symptoms and academic performance. For this demonstration, we analyzed mother and father reports of the nine ADHD-inattention symptoms at the first time point [*N =* 752].^2^

### Measures

The measure used was the ADHD-inattention subscale of the Child and Adolescent Disruptive Behavior Inventory (Burns and Lee, 2010a,b). The inattention (ADHD-IN) subscale consisted of the nine inattention symptoms. Symptoms of inattention were rated on a six-point scale ranging from 0 (*nearly occurs none of the time* [e.g., 2 or fewer times per month]) to 5 (*nearly occurs all the time* [e.g., many times per day]). ADHD-IN symptoms were split into three parcels of three items each, using a parceling technique designed to create homogeneous parcels (Little et al., 2013).^3^ The parcels were treated as continuous measures. Skewness and kurtosis of the parcels was moderate (Skewness was between 1.12 and 2.08 for all parcels; kurtosis was between 0.79 and 3.92 for all parcels; skewness and kurtosis were significantly different from 0 for all observed variables). Previous simulations have shown that LMS is robust to this level of non-normality (Harring et al., 2012).

### Analysis Strategy

In the first step, we fit the basic TMU model to the data. Subsequently, the standard LD and LM models with only linear trait-method relationships were examined. In the final step, we tested the new quadratic extensions of the LD and LM models. The fit of the quadratic models was compared against the fit of the linear models using a likelihood ratio test. Statistical significance of the quadratic term was determined using the likelihood ratio test as opposed to a Wald test based on the model-estimated standard errors, as recommended by Klein and Moosbrugger (2000).

### Results

Table 1 shows descriptive statistics for the mother and father ratings of child inattention. We first estimated a basic TMU model with strong measurement equivalence (equal intercepts loadings) across methods. The model also included correlated measurement error variables between identical parcels across methods to account for relationships between the indicators that are not accounted for by the trait factor (Figure 1A with correlated residuals added; Marsh and Hocevar, 1988). The TMU model showed a good fit to the data when parcel-specific effects were accounted for, χ^2^(9,N = 752) = 17.42,p = 0.04, RMSEA = 0.04, CFI = 0.998. Table 2 shows the estimated model parameters.

**Table 1:** Correlation matrix and descriptive statistics of mother and father ratings of child inattention from Burns et al. (2014) study.

Variable	1	2	3	4	5	6
(1) Mother, Parcel 1	—					
(2) Mother, Parcel 2	0.87	—				
(3) Mother, Parcel 3	0.82	0.85	—			
(4) Father, Parcel 1	0.77	0.74	0.72	—		
(5) Father, Parcel 2	0.71	0.78	0.72	0.88	—	
(6) Father, Parcel 3	0.69	0.72	0.78	0.83	0.86	—

*M*	0.94	1.20	1.10	0.96	1.18	1.11
*SD*	0.96	1.13	1.05	0.94	1.12	1.08
Skewness	1.41	1.22	1.39	1.20	1.12	1.27
Kurtosis	1.89	1.11	1.89	1.26	0.81	1.39

**Table 2:** Parameter estimates from the basic TMU model fit to mother and father ratings of child inattention.

Parameter	Estimate (standardized estimate)	*SE*	*p*
E(Inattention_Mother_)	0.954	0.034	<0.001
E(Inattention_Father_)	0.977	0.035	<0.001
V ar(Inattention_Mother_)	0.759	0.046	<0.001
V ar(Inattention_Father_)	0.761	0.049	<0.001
Cov(Inattention_Mother_, Inattention_Father_)	0.637 (0.84)	0.042	<0.001
λ_11_	1 (0.91)	—	—
λ_21_	1.23 (0.95)	0.02	<0.001
λ_31_	1.09 (0.90)	0.02	<0.001
λ_12_	1 (0.93)	0.02	<0.001
λ_22_	1.23 (0.95)	0.02	<0.001
λ_32_	1.09 (0.90)	0.02	<0.001
_α_1__	0	—	—
_α_2__	0.03	0.03	0.41
_α_3__	0.09	0.03	0.008
V ar(𝜀_11_)	0.13 (0.17)	0.01	<0.001
V ar(𝜀_21_)	0.10 (0.10)	0.01	<0.001
V ar(𝜀_31_)	0.21 (0.19)	0.02	<0.001
V ar(𝜀_12_)	0.13 (0.15)	0.01	<0.001
V ar(𝜀_22_)	0.12 (0.10)	0.02	<0.001
V ar(𝜀_32_)	0.22 (0.19)	0.02	<0.001
Cov(𝜀_11_,𝜀_12_)	0.06 (0.43)	0.01	<0.001
Cov(𝜀_21_,𝜀_22_)	0.03 (0.32)	0.01	<0.001
Cov(𝜀_31_,𝜀_32_)	0.09 (0.44)	0.01	<0.001

*λ_i_ = factor loadings of indicators onto latent TMU factors; α_i_ = intercepts; 𝜀_*im*_ = measurement error variable; *i =* indicator; *m =* method (1 = mother report, 2 = father report)*.

#### Measurement Model

Standardized factor loadings for the observed inattention indicators are presented in Table 2, and represent the correlation between the observed variable and the latent TMU factor. Reliability is indicated by the squared standardized loadings. The lowest standardized loading was 0.9, and the highest was 0.95. The reliabilities of the observed indicators thus ranged from 0.81 to 0.9. The correlations between measurement error variables pertaining to identical parcels across methods ranged between 0.32 and 0.44, indicating that some amount of parcel-specific variance was shared across mother and father reports. The mother and father TMU factors were strongly correlated (ϕ = 0.84), indicating strong convergent validity across parent reports.

#### LD Model

In the LD analyses, mother report of child inattention was selected as the reference method (*m* = 1), against which father report of child inattention (*m* = 2) was contrasted. Mother report was selected as the reference method because previous research has suggested that mental health professionals tend to view mothers as most knowledgeable about children’s mental health symptoms (Loeber et al., 1990). As a consequence, the trait factor *T*_1_ in the model represented inattention true scores as measured by mother reports and the method factor *M*_2_ represented the difference between true father and true mother reports.

The model was parameterized with the trait-method relationship as a linear regression of the method factor on the trait factor (Figure 1B). The key parameters in the linear LD model are the mean and variance of the trait factor and the parameters in the linear regression of the method factor on the trait factor. The mean of the reference trait factor was estimated to be 0.95, meaning that the average mother rating of inattention symptoms was relatively low (given the range of the response scale from 0 to 5). The variance of the mother trait factor was estimated to be 0.69.

The relationship between mother-rated inattention and the method effect of father ratings was parameterized as a linear regression. The following structural regression equation was estimated:

$$M_{2}=0.18-0.16T_{1}+{\text{ζ}}_{2}.$$

**FIGURE 3:**
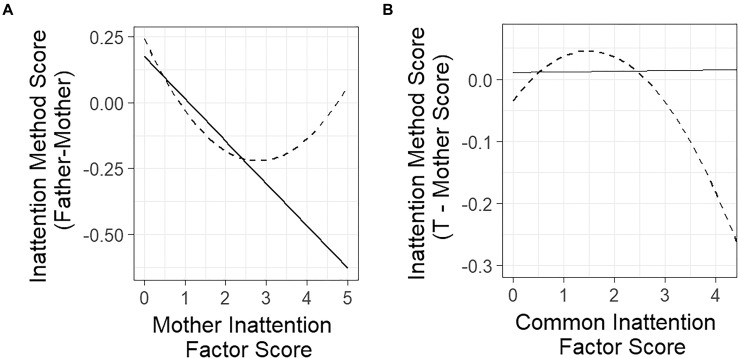
Plots of the estimated linear and quadratic trait-method relationships in LD and LM models for parent ratings of children’s inattention. **(A)** LD model. **(B)** LM model. Relationships estimated with linear models are represented as solid lines. Relationships estimated with quadratic models are represented as dashed lines.

Notice that the slope parameter (β_12_ = –0.16) was significant (*z* = –5.98) and negative (the standardized regression coefficient, which equals the correlation between *T*_1_ and *M*_2_, was estimated to be -0.28, indicating a medium size effect). This linear relationship is illustrated in Figure 3A. As can be seen in the figure, the negative relationship in this example indicated that for lower trait scores of inattention (as rated by mothers), the discrepancy between mother and father reports was small (difference scores close to zero). As mother trait scores increased, the difference scores tended to become more and more negative (indicating that the discrepancy between mother and fathers increased). Specifically, based on the estimated negative slope coefficient, the father–mother rating difference scores were expected to become smaller by 0.16 points for every one point increase in mother-rated inattention symptoms. This indicated that fathers’ underestimation of inattention symptoms relative to mother reports was stronger at higher levels of inattention than for lower inattention scores (This can in part be explained by a floor effect, because the scale used in this example is bound by zero).

The model-implied mean of the method factor can be derived from the regression equation as *E*(*M*_2_) = β_02_ + β_12_*E*(*T*_1_) = 0.02. This indicated that on average, the discrepancy between true mother and true father reports in this application was close to zero. The model-implied variance of the method factor is given by V ar(M_2_) = ${\text{β}}_{12}^{2}$V ar(T_1_) + V ar(ζ_2_) = 0.25. The residual variance V ar(ζ_2_) was estimated to be 0.23, showing that the method factor variance was only slightly reduced by taking into account the regression on the trait factor (*R*^2^ = 0.08).

In summary, the linear model suggested that the mother and father ratings of inattention showed the highest agreement at the low end of the scale, and that father and mother ratings became increasingly discrepant as mother-rated levels of inattention increased. For higher levels of inattention, fathers tended to more strongly underestimate inattention symptoms relative to mothers according to the linear model.

A likelihood ratio test comparing the linear LD model to an LD model with a quadratic term in the regression of the method factor on the trait factor fit the data significantly better than the linear model, χ^2^(1,N = 752) = 6.682,p = 0.009. This indicated that the quadratic term was statistically significant. The estimated quadratic structural regression equation was M_2_ = 0.24 - 0.33T_1_ + 0.06$T_{1}^{2}$ + ζ_2_. The quadratic coefficient was significant (*z =* 2.93) and positive, indicating a u-shaped curve. Figure 3A shows the model-implied quadratic regression compared to the linear model. The roots of the equation were 0.87 and 4.74, meaning that the model-estimated points at which the estimated value of the *M*_2_ was 0 were at 0.87 and 4.74. The first root is very close to the mean of the trait scores, meaning that when mothers rated inattention at the average level, fathers tended to agree on the level of inattention. The second root is at the extreme end of the possible range of observed scores, indicating that when mothers rated inattention very highly, fathers tended to agree on the level of inattention as well.

The inflection point of the function was 3.1, meaning that the slope of the line was decreasing when mother-rated inattention symptoms were below 3.1, and increasing when mother-rated inattention symptoms were above 3.1. The minimum value of *M*_2_ when mothers rated inattention at 3.1 was -0.21, indicating that the highest discrepancy between mothers and fathers was at an intermediate level of inattention symptoms, and that the level of discrepancy was relatively small.

The quadratic function showed a substantial difference from the linear model. The quadratic model suggested that fathers were *not* very discrepant from mothers at either *low or high* levels of mother-rated inattention. However, at intermediate levels of mother-rated inattention (between mother ratings of 1 and 4), father ratings tended to be lower than mother ratings. The quadratic model therefore showed that the discrepancy between the two methods at higher levels of the trait (above 3.1), is just as small as it is at lower levels of the trait, which is a characteristic that would have been overlooked had we only fit the linear model.

#### LM Model

The LM model is an alternative parameterization of the basic TMU model, and thus showed an equivalent fit to the LD model for this data when only the linear trait-method relationship was specified. The structural equations for the TMU factors were set up as follows:

$$\begin{array}{l} T_{1}=T-M_{2}^{*}(\text{mothers})\\ T_{2}=T+M_{2}^{*}(\text{fathers}), \end{array}$$

where *T*_1_ is the TMU factor for the mother ratings of child inattention, and *T*_2_ is the TMU factor for the father ratings of child inattention. As such, the method factor for fathers ($M_{2}^{*}$) was retained and the mother TMU factor *T*_1_ was assigned a loading of -1 on the method factor.

In the linear LM model, *E*(*T*) was estimated to be 0.93 and *Var*(*T*) was estimated to be 0.64. This reflects the fact that the trait factor *T* in the LM model reflects the average of both the mother and the father TMU variables, instead of only the reference variable for mothers.

The model-estimated linear regression equation for $M_{2}^{*}$ was $M_{2}^{*}$ = 0.011+0.001T +$\zeta_{2}^{*}$. Neither the intercept nor the slope coefficient were significant, *z =* 0.04 and 0.621, respectively. This means that no significant linear relationship was found between *T* and $M_{2}^{*}$ in the data. In other words, the mother and father deviations from the average inattention scores were not linearly related to the average. In addition, the model-implied mean of the method factor was not significantly different from zero. The residual variance was estimated to be Var($\zeta_{2}^{*}$) = 0.06 and was significant (*z =* 13.08).

The quadratic extension to the LM model fit the data better than the linear model as shown by a likelihood ratio test comparing the two models,χ^2^(1,N = 752) = 9.614,p = 0.002. The model-estimated structural equation was $M_{2}^{*}$ = -0.034 + 0.11T - 0.036T^2^ + $\zeta_{m}^{*}$. The quadratic coefficient was significant (*z* = -2.92) and negative, indicating an inverse u-shaped curve. Figure 3B displays both the linear and quadratic functions for the LM analyses.

The roots of the quadratic function were 0.35 and 2.7, meaning that at these inattention trait values, the model-implied value of the method factor was zero. The inflection point of the model-estimated function was 1.78, meaning that for inattention trait factor scores below 1.53, the overall slope of the line was positive, and for trait scores greater than 1.53, the overall slope of the line was negative. The model-predicted maximum value of $M_{2}^{*}$ was 0.05, which means that when the mean level of mother and father ratings was 1.53, the average difference between mother and father scores was 0.1. Above trait values of 2.7, the model-estimated value of the method effect became increasingly negative. At the value of the highest estimated trait factor score in the data (*T =* 4.2), the model-estimated value of $M_{2}^{*}$ was -0.21, meaning that the difference between mother and father ratings was estimated to be 0.42, with mothers rating higher than fathers. At this level of the scale, this is almost half the difference between a rating of 4 (anchor is “*Very often occurs [several times per day]”*) and 5 (anchor is “*Nearly occurs all the time [e.g., many times per day]”*). Because the mother report’s method factor loadings were fixed to -1, this means that mothers rated children higher on inattention than fathers at this level of the common inattention trait.

In summary, each quadratic model provided substantively different information than the linear models, showing that the quadratic model may be useful in identifying trait levels at which convergent validity is stronger or weaker. Both the quadratic LD and LM models revealed that convergent validity was strongest at low levels of inattention, and marginally weaker at higher levels of inattention. When comparing the father–mother discrepancy to mother ratings of inattention, discrepancies were highest at intermediate levels of inattention. When comparing the method discrepancy to the mean of mother and father-rated inattention, discrepancies were highest at high levels of inattention.

## Simulation Study

In addition to the practical application, we examined the performance of the quadratic LD and LM models under a larger set of conditions using a Monte Carlo simulation (The details of the simulations are reported in Appendix B). Although the LMS estimation procedure has been well-studied (Klein and Moosbrugger, 2000; Kelava et al., 2011; Harring et al., 2012), little is known about issues of statistical power for common sample sizes in psychological research when using the LD and LM models with quadratic effects. To test power, the simulation used parameter estimates from the LD and LM models presented in the application as population models and varied the sample size, indicator reliability, and effect size of the quadratic term in both the LM and LD models. Effect size values were based on Cohen’s recommendations for small, medium, and large partial regression coefficients (Cohen, 1992).

To test the Type 1 error rate, we used parameter estimates from the LD and LM models as population models, but changed the value of the quadratic term to zero. The estimator then searched for a quadratic effect where none was present, and the proportion of replications with a significant quadratic effect was regarded as the Type I error rate. The Type 1 error models were varied with respect to sample size and indicator reliability. Further details of the simulation and software code for the simulation are provided in Appendix B and the online Supplementary Material.

The power simulation found that for medium and large effect sizes, with indicator reliability of 0.8, power of 0.8 was achieved at sample sizes above *N =* 250. Small effect sizes resulted in low power at all sample sizes and reliabilities. Similar results were found for the LM model, although the LM model overall had slightly reduced power compared to the LD model. The Type 1 error rates were within the acceptable range (between 2.5 and 7.5%) for all conditions except *N =* 100, where there was slight inflation of the type I error rate (actual α = 10%). The findings are in line with previous simulations with the LMS estimator (Klein and Moosbrugger, 2000; Kelava et al., 2011; Cham et al., 2012; Harring et al., 2012), which found that it performs better with larger effect sizes, and that power to detect latent interactions is frequently lower than would be expected in an observed variable model.

## Discussion

The LD and LM models of CFA-MTMM analysis both allow examining linear relationships between trait and method factors. In the present paper, we proposed extensions of both models to incorporate potential non-linear relationships between traits and methods. The extensions were shown to provide useful insights into the convergent validity of methods and to perform well both in an empirical application and a Monte Carlo simulation study.

The LD model requires the choice of a reference method. A method factor represents the difference between a given non-reference trait and the trait pertaining to the reference method. The LM model does not require the choice of a reference method. Instead, a common trait factor is defined as the grand mean of all method-specific traits and method factors represent the deviation of a method-specific trait from the common trait.

The quadratic LD model represents the potentially non-linear effects of a reference trait on the discrepancies between each non-reference method and the reference method. The quadratic LM model represents the potentially non-linear effects of a common trait on the method-specific deviations from that common trait. The LD model is more appropriate when researchers are able to specify a clear reference method that is a gold standard method or has a clear structural difference from the other methods. The LM model is more appropriate when researchers do not have a clear reference method available and when the grand mean across method-specific traits is meaningful as a common trait represents a meaningful trait score.

In our example of mother and father reports of inattention, a case could be made for either model. If mother reports were seen as a clear “gold standard” in the study of child inattention symptoms (e.g., following Loeber et al., 1990), the LD model (with mother reports as reference method) may be preferred and the true scores based on mother reports would be used as “best estimates” of the children’s inattention trait values. If instead a researcher views parents as more or less interchangeable sources of information, the average across mother and father reports may be seen as a more meaningful trait score, and the LM model may be chosen. Regarding the statistical performance of the quadratic LD and LM models, both appear to work similarly well to detect quadratic effects. Our simulation revealed that small effects are difficult to detect in both models.

Our work extends and complements Koch et al. (2017) paper, who examined LD models and CTC(M-1) models in which the method effect variables were regressed on explanatory variables that interacted with the level of the trait variable. These models were also estimated using the LMS method.

### Advantages of the Quadratic LD and LM Models

The LD and LM models both have their roots in classical test (true score) theory. As a result, they represent CFA-MTMM models that are based on explicit mathematical definitions of latent trait and method variables. Both models allow researchers to examine relationships between trait levels and method effects and these relationships have a clear meaning.

The new linear and quadratic LD and LM models both allow researchers to examine the extent to which method effects are dependent on trait levels. The new quadratic models allow for less restrictive assumptions about the trait-method relationship (i.e., the relationship does not have to be linear as in the standard LD and LM models). These models therefore allow researchers to examine more complex questions about method effects than were possible with previous latent variable MTMM modeling techniques. Our simulations showed that the estimation of quadratic effects in the LD and LM models using the LMS method appears to have sufficient statistical power at sample sizes above 250 with medium to large quadratic effects. Both models also showed acceptably low Type-1 error rates.

### Limitations of the Quadratic LD and LM Models

Limitations of the LD and LM models are that they require strong measurement invariance of the indicators across methods for a meaningful interpretation. That is, the LD scores used to define method factors only have meaning when the latent method-specific trait factors *T_m_* representing each TMU are measured with the same origin and units of measurement. Otherwise, the difference scores represent a mix between true method discrepancies and measurement-related differences between methods. This is a limiting factor for many research situations, especially ones that do not use the same measurement instruments (e.g., questionnaires) across methods.

A limitation of the quadratic extensions to the LD and LM models is that the statistical power to detect small quadratic effects is very low for all sample sizes. Researchers may therefore have difficulty detecting such effects unless they use extremely large samples, which may not be feasible in many MTMM studies. An additional limitation is that we studied only the LMS method for parameter estimation in this paper. Future studies should examine alternative non-linear SEM estimation methods as they may provide greater statistical power.

An additional limitation of the quadratic LD and LM models is that they assume that the discrepancy between methods is such that one method consistently rates higher or lower at a given level of the trait. However, the *absolute values* of a method factor may be of greater interest in practice, because both large positive and small negative method factor scores indicate strong discrepancies between methods (i.e., large method effects). It may be the case that the absolute values of the method factor are dependent on the level of the underlying trait or some other explanatory variable. In this case, the absolute value of the method factor should be the subject of study, disregarding the direction of the discrepancy. Further research should examine ways to model the absolute value of the method factor in addition to the observed values of the method factor. Future studies may also examine non-linear relationships between method factors and external variables. Finally, we recommend that researchers who use the techniques presented here should make an effort to replicate findings of quadratic trait-method relationships with fresh data, especially when such analyses are exploratory in nature rather than theory-driven.

## Conclusion

The LD and LM models represent an important advancement in the modeling of multitrait-multimethod data. Along with other new models for examining trait-method interactions (e.g., Litson et al., 2017), they represent a new avenue for multitrait-multimethod research that explicitly examines the relationship between traits and methods and asks questions about convergent validity that are innovative and important. The quadratic extensions of the LD and LM models expand the toolbox of multimethod researchers, as they make it possible to examine more complex trait-method relationships. Such complex relationships may reveal practically meaningful differences in the level of convergent validity across trait levels.

## Author Contributions

FH and CG wrote the majority of the manuscript. GB and MS collected the data used in the application and provided some comments on the manuscript.

## Conflict of Interest Statement

The authors declare that the research was conducted in the absence of any commercial or financial relationships that could be construed as a potential conflict of interest.

## Footnotes

1 This is only true for the two-method case. Designs with three or more methods require that *m* – 1 method factors be estimated. The value of the last method effect is a deterministic function of the others (Pohl and Steyer, 2010).

2 In an actual substantive application, the nested data structure (children nested within schools) should be taken into account, for example, by using a robust sandwich estimator. Given that our analyses serve illustrative purposes only, we did not use this approach here.

3 Item parceling rests on the assumption of unidimensionality of the items, which is a strong assumption that should be thoroughly investigated in item level analyses prior to creating parcels. For a thorough discussion of the pros and cons of item parceling (see Little et al., 2002).
