# Supplementary material for: Examining Quadratic Relationships Between Traits and Methods in Two Multitrait-Multimethod Models
Source: Front Psychol. 2019 Mar 14;10:353. doi: 10.3389/fpsyg.2019.00353 (PMC6426770; doi:10.3389/fpsyg.2019.00353)
Supplement: Supplementary file 1 [file Data_Sheet_1.PDF]

Appendix A

Identification Of Structural Parameters For The LD And LM Models

In this appendix, we derive the identification formulas for each structural variance parameter of both the LD and LM models and compare them to each other. We use the rules of covariance algebra as described by Kenny (1979) to derive the variance of the trait and method factors and the covariance between the trait and method factors in both the LD and LM models.

Identification Of LD Model Parameters

The variance of the trait factor for the LD model is the variance of $T_{1,}$ because T_1 is defined as the trait factor. The variance of the method factor is identified as a function of the two underlying TMU variables. We can start by examining the definition of the method factor in the LD model, which is given by:

$$M = T_2 - T_1 \qquad \text{A1}$$

Following from equation A1, the variance of the method factor can be written as follows:

$$Var(M) = Var(T_2 - T_1) \qquad \text{A2}$$

Using the variance definition and sum rules of covariance algebra (Kenny, 1979, p.21), equation A2 becomes :

$$\begin{aligned} Var(M) &= Cov(T_2 - T_1, T_2 - T_1) \\ Var(M) &= Cov(T_2, T_2) + Cov(T_2, -T_1) + Cov(-T_1, T_2) + Cov(-T_1, -T_1) \\ Var(M) &= Var(T_1) + Var(T_2) + 2Cov(-T_1, T_2) \end{aligned} \qquad \text{A3}$$

Finally, following the constant rule of covariance algebra (Kenny, 1979, p.21), equation A3 becomes :

$$Var(M) = Var(T_1) + Var(T_2) - 2Cov(T_2, T_1) \tag{A4}$$

The variance of the method factor in the LD model is therefore identified by the sum of the variances of the two TMU factors and the covariance between the TMU factors.

In the LD model the covariance between the trait and method factors is identified by a function of the covariance of the two TMU factors and the variance of the reference factor. This follows from substituting the definition of the trait and method factors into the covariance formula:

$$Cov(T_1, M) = Cov[T_1, (T_2 - T_1)] \quad . \tag{A5}$$

Following the sum rule, equation A5 becomes :

$$Cov(T_1, M) = Cov(T_1, T_2) + Cov(T_1, -T_1) \tag{A6}$$

Following the constant rule and variance definition rules, equation A6 become :

$$Cov(T_1, M) = Cov(T_1, T_2) - Var(T_1) \tag{A7}$$

Therefore the covariance of T_1 and M in the LD model is a function of the covariance between T_1 and T_2 and the variance of T_1.

Identification of LM model parameters.

Note that the following identification formulas are only true when the number of methods compared is two. More complex identification formulas are required when using three or more methods with the LM model.

The identification for the variance of the trait factor follows from the definition of the trait factor for the LM model when there are two methods:

$$T = \frac{T_1 + T_2}{2}$$
$$T = \frac{1}{2}T_1 + \frac{1}{2}T_2$$
$$Var(T) = Var(\frac{1}{2}T_1 + \frac{1}{2}T_2) \quad . \qquad \text{A8}$$

Following from the variance definition and sum rules of covariance algebra, equation A8 becomes:

$$Var(T) = Cov(\frac{1}{2}T_1 + \frac{1}{2}T_2, \frac{1}{2}T_1 + \frac{1}{2}T_2)$$
$$Var(T) = Cov(\frac{1}{2}T_1, \frac{1}{2}T_1) + Cov(\frac{1}{2}T_1 + \frac{1}{2}T_2) + Cov(\frac{1}{2}T_2, \frac{1}{2}T_1) + Cov(\frac{1}{2}T_2, \frac{1}{2}T_2)$$
$$Var(T) = Var(\frac{1}{2}T_1) + Var(\frac{1}{2}T_2) + 2Cov(\frac{1}{2}T_1, \frac{1}{2}T_2) \quad . \qquad \text{A9}$$

And finally, following the Constant rule of covariance algebra, equation A9 becomes :

$$Var(T) = \frac{1}{2} \bullet \frac{1}{2} Var(T_1) + \frac{1}{2} \bullet \frac{1}{2} Var(T_2) + 2 \bullet \frac{1}{2} \bullet \frac{1}{2} Cov(T_1, T_2)$$
$$Var(T) = \frac{1}{4} Var(T_1) + \frac{1}{4} Var(T_2) + \frac{2}{4} Cov(T_1, T_2)$$
$$Var(T) = \frac{Var(T_1) + Var(T_2) + 2Cov(T_1, T_2)}{4} \qquad \text{A10}$$

Equation A10 shows that the variance of the LM model is a function of both TMU variances and their covariance.

In the LM model, the variance of the method factor can be derived from the definition of the method factor:

$$M^* = T_2 - T$$
$$M^* = T_2 - (\frac{1}{2}T_1 + \frac{1}{2}T_2)$$
$$Var(M^*) = Var[T_2 - (\frac{1}{2}T_1 + \frac{1}{2}T_2)]$$

A11

Following the sum rule, equation A11 becomes:

$$Var(M^*) = Cov[T_2 - (\frac{1}{2}T_1 + \frac{1}{2}T_2), T_2 - (\frac{1}{2}T_1 + \frac{1}{2}T_2)]$$
$$Var(M^*) = Cov(T_2, T_2) + Cov[T_2, -(\frac{1}{2}T_1 + \frac{1}{2}T_2)] + Cov[-(\frac{1}{2}T_1 + \frac{1}{2}T_2), T_2] + Cov[-(\frac{1}{2}T_1 + \frac{1}{2}T_2), -(\frac{1}{2}T_1 + \frac{1}{2}T_2)]$$
$$Var(M^*) = Var(T_2) + Cov(T_2, -\frac{1}{2}T_1) + Cov(T_2, -\frac{1}{2}T_2) + Cov(-\frac{1}{2}T_1, T_2) + Cov(-\frac{1}{2}T_2, T_2) + Cov(-\frac{1}{2}T_1, -\frac{1}{2}T_1) +$$
$$Cov(-\frac{1}{2}T_1, -\frac{1}{2}T_2) + Cov(-\frac{1}{2}T_2, -\frac{1}{2}T_1) + Cov(-\frac{1}{2}T_2, -\frac{1}{2}T_2)$$

A12

Following the constant rule, equation A12 becomes

$$Var(M^*) = Var(T_2) - \frac{1}{2}Cov(T_2, T_1) - \frac{1}{2}Cov(T_2, T_2) - \frac{1}{2}Cov(T_1, T_2) - \frac{1}{2}Cov(T_2, T_2) +$$
$$\frac{1}{4}Cov(T_1, T_1) + \frac{1}{4}Cov(T_1, T_2) + \frac{1}{4}Cov(T_2, T_1) + \frac{1}{4}Cov(T_2, T_2)$$

A13

Cancelling redundant terms leads to:

$$Var(M^*) = \frac{1}{4}Var(T_1) + \frac{1}{4}Var(T_2) - \frac{1}{2}Cov(T_1, T_2)$$
$$Var(M^*) = \frac{Var(T_1) + Var(T_2) - 2Cov(T_1, T_2)}{4}$$

A14

Therefore, in the LM model, the variance of the method factor is also identified by a function of the difference in variance between the TMU factors and the covariance between them.

The identification of the covariance between trait and method factors can be derived by substituting the definitions of the trait and method factors and using covariance algebra:

$$Cov(T, M^*) = Cov[(\frac{1}{2}T_1 + \frac{1}{2}T_2), (T_2 - (\frac{1}{2}T_1 + \frac{1}{2}T_2))] \quad \text{A15}$$

From equation A15, applying the sum rule gives:

$$\begin{aligned} Cov(T, M^*) &= Cov[(\frac{1}{2}T_1 + \frac{1}{2}T_2), T_2] + Cov[(\frac{1}{2}T_1 + \frac{1}{2}T_2), -(\frac{1}{2}T_1 + \frac{1}{2}T_2)] \\ Cov(T, M^*) &= Cov(\frac{1}{2}T_1, T_2) + Cov(\frac{1}{2}T_2, T_2) + Cov(\frac{1}{2}T_1, -\frac{1}{2}T_1) + \\ &\quad Cov(\frac{1}{2}T_1, -\frac{1}{2}T_2) + Cov(\frac{1}{2}T_2, -\frac{1}{2}T_1) + Cov(\frac{1}{2}T_2, -\frac{1}{2}T_2) \end{aligned} \quad \text{A16}$$

From equation A16, applying the constant rule and cancelling out redundant terms gives:

$$\begin{aligned} Cov(T, M^*) &= \frac{1}{2}Cov(T_1, T_2) + \frac{1}{2}Var(T_2) - \frac{1}{4}Var(T_1) - \frac{1}{4}Cov(T_1, T_2) - \frac{1}{4}Cov(T_2, T_1) - \frac{1}{4}Var(T_2) \\ Cov(T, M^*) &= \frac{1}{4}Var(T_2) - \frac{1}{4}Var(T_1) \\ Cov(T, M^*) &= \frac{Var(T_2) - Var(T_1)}{4} \end{aligned} \quad \text{A17}$$

Therefore, in the LM model, the covariance between the trait and method factors is identified by a function of the variances of each of the trait method units, when the method loadings for T_1 are negative and the method loadings for T_2 are positive. The identification of each portion of the structural model for the LM and LD models from the parameters of the TMU model is shown in Table A1.

Table A1.
Identification of structural variance parameters from TMU model for LD and LM models

Parameter	Notation in LD model	Identification in LD model	Notation in LM model	Identification in LM model
Variance of trait variable	$Var(T_1)$	$Var(T_1)$	$Var(T)$	$\frac{Var(T_1)+Var(T_2)+2Cov(T_1,T_2)}{4}$
Variance of method variable	$Var(M)$	$Var(T_1)+Var(T_2)-2Cov(T_2,T_1)$	$Var(M^*)$	$\frac{Var(T_1)+Var(T_2)-2Cov(T_1,T_2)}{4}$
Covariance of trait and method variable	$Cov(T_1,M)$	$Cov(T_1,T_2)-Var(T_1)$	$Cov(T,M^*)$	$\frac{Var(T_2)-Var(T_1)}{4}$

Note. LD=Latent Difference; LM=Latent Means; TMU=Trait-method unit; T_m =Trait-method unit for method *m*; *M* =Method variable for the LD model; *T*=Trait variable for the LM model; M^*=Method variable for the LM model.

Appendix B

Simulation Study Examining Statistical Power and Type 1 Error Rates

For the Quadratic LD and LM Models

A Monte Carlo study examining the power and type 1 error rates of the LMS method to detect quadratic effects in LD and LM models was performed. We attempted to address the following specific questions: 1) Under which conditions does the LMS method for the quadratic LD and LM methods provide sufficient statistical power for the effect sizes observed in the applications? 2) Do Type-1 errors occur only 5% of the time in the quadratic LD or LM models?

Data Generation

The simulation used parameter values taken from the two sets of applications that showed significant quadratic effects. Separate population models were simulated for each type of model (LD and LM), because quadratic trait-method relationships in one model do not necessarily imply similar quadratic trait method relationships in the other. Three different factors were varied in the simulation: the sample size (14 levels), reliability of indicators (4 levels), and the amount of residual variance in the structural part of the model [$Var(\zeta_{M_2})$ and $Var(\zeta_{M^*})$] (5 levels). The sample sizes ranged from N = 100 to N = 750, in increments of 50. The reliability of indicators ranged from .6 to .9, representing typical values of indicator reliability in the social sciences. The size of the quadratic parameter was selected to represent small, medium, and large effect sizes for partial correlation coefficients (.02, .15, .35) according to Cohen (1980). The implied residual variance was calculated using formulas for the variance of product terms from Goodman (1960). To examine type 1 error, we also simulated an population model with no quadratic effect present for each of the sample size and reliability conditions. Using all

combinations of the data conditions resulted in 224 population models for the latent difference and latent means models. The R package MplusAutomation was used to generate the models (Hallquist & Wiley, 2017; see the appendix for code used to generate models).

Analysis Strategy

Each simulated dataset was fit with both a misspecified linear model and a quadratic model. We recorded instances of non-convergence and improper solutions. The log-likelihood values of the misspecified linear models were then compared to the log-likelihood values of the appropriately specified quadratic models to test the statistical significance of the quadratic effect with the likelihood ratio test. To answer the proposed questions, power, and type I error were analyzed and summarized for each cell of the simulation design, and compared against typical cutoffs of acceptability.

Power

Both power and type 1 error for the quadratic effects were examined using the chi-square likelihood ratio test suggested by Klein and Moosbrugger (2000) for the LMS method. For models that contained a true quadratic effect, power was analyzed as the percentage of replications that showed a significant value of the likelihood ratio test statistic or the wald test. Power was considered acceptable when 80% of replications with a true effect in the population correctly rejected the null hypothesis that there was no quadratic effect (Cohen, 1980).

Type I error

For models that contained no true quadratic effect, Type-1 error was analyzed as the percentage of replications that showed a significant value of the likelihood ratio test statistic despite a true value of zero in the population. Bradley (1978) suggested a liberal criterion for

Type-I error, in which Type-I error rates between 2.5% and 7.5% are considered acceptable for an α level of 5%. This criterion was adopted in the present study.

Results

Convergence and Improper solutions

All replications converged on a solution. Improper solutions were a problem in less than 0.01% of replications. A detailed analysis showed that improper solutions only occurred for the smallest sample size of N=100 and reliability of .6 in the LD model with small and medium effect sizes (.06%, and .02% of replications, respectively). In the LM model, improper solutions also only occurred for N=100 and reliability of .6, with small, medium, and large effect sizes (.04%, .06%, and .01%, respectively). In both cases, the improper estimate was a latent variable correlation greater than 1. These replications were included in the data, since the occurrence of improper solutions was so small as not to influence the overall conclusions of the study.

Power

Power was evaluated for the likelihood ratio test and the wald test. Power for the wald test and likelihood ratio tests were approximately equal. A t-test comparing obtained power values for each cell showed that the difference between the two tests was not significant, $t(165)=-.32$, $p=.74$. Power was substantially affected by effect size, sample size, and indicator reliability. Figure A1 shows the power for the LM and LD models by sample size, effect size and indicator reliability. Power of .8 was attained for large effects and indicator reliability with sample sizes greater than 250. For medium-sized effects, power of .8 was attained with sample sizes greater than 450. For small effects, power was below .5 for all sample sizes with low and high indicator reliability. Power was slightly lower for the LM models overall with both tests, and low reliability more strongly affected power in the LM model.

Type I error

For the LD and LM model, the type I error rates for the LRT were between 2.5% and 7.5% for all data conditions. For the wald test, the type I error rate for both the LM and LD models were 10% only when indicator reliability was .8 and sample size was N=100. In all other conditions, type I error rates were between 2.5% and 7.5%.

Discussion

The simulation found that estimating the quadratic LD and LM models works well with the LMS method. The power for medium and large effect sizes is good when sample sizes are in the range typically seen in psychological research (between N=250 and N=500). The power for small effect sizes is low to moderate with all sample sizes. The LM model has slightly lower power to detect quadratic effects than the LD model. This is likely because the method factor in the LM Models were much smaller, and the quadratic effects were also substantially smaller in absolute value, although the standardized effect sizes were equivalent to the LD model.

Type I error was appropriate for all conditions, suggesting that the quadratic models do not simply fit extraneous noise in the models, and a significant result usually indicate a true effect in the population.

Models examining interaction and higher order effects that correct for measurement error typically suffer from low statistical power, and previous findings of simulations examining the LMS estimator show low power for latent interaction effects (Cham et al., 2012; Kelava et al., 2012). An unfortunate consequence of this is that in multimethod research, quadratic relations between trait and method may be inappropriately ignored. Further research should examine alternative estimation strategies and approaches (e.g., latent variable mixture modeling,

estimation techniques for small samples) to be able to detect quadratic trait-method relationships more often.

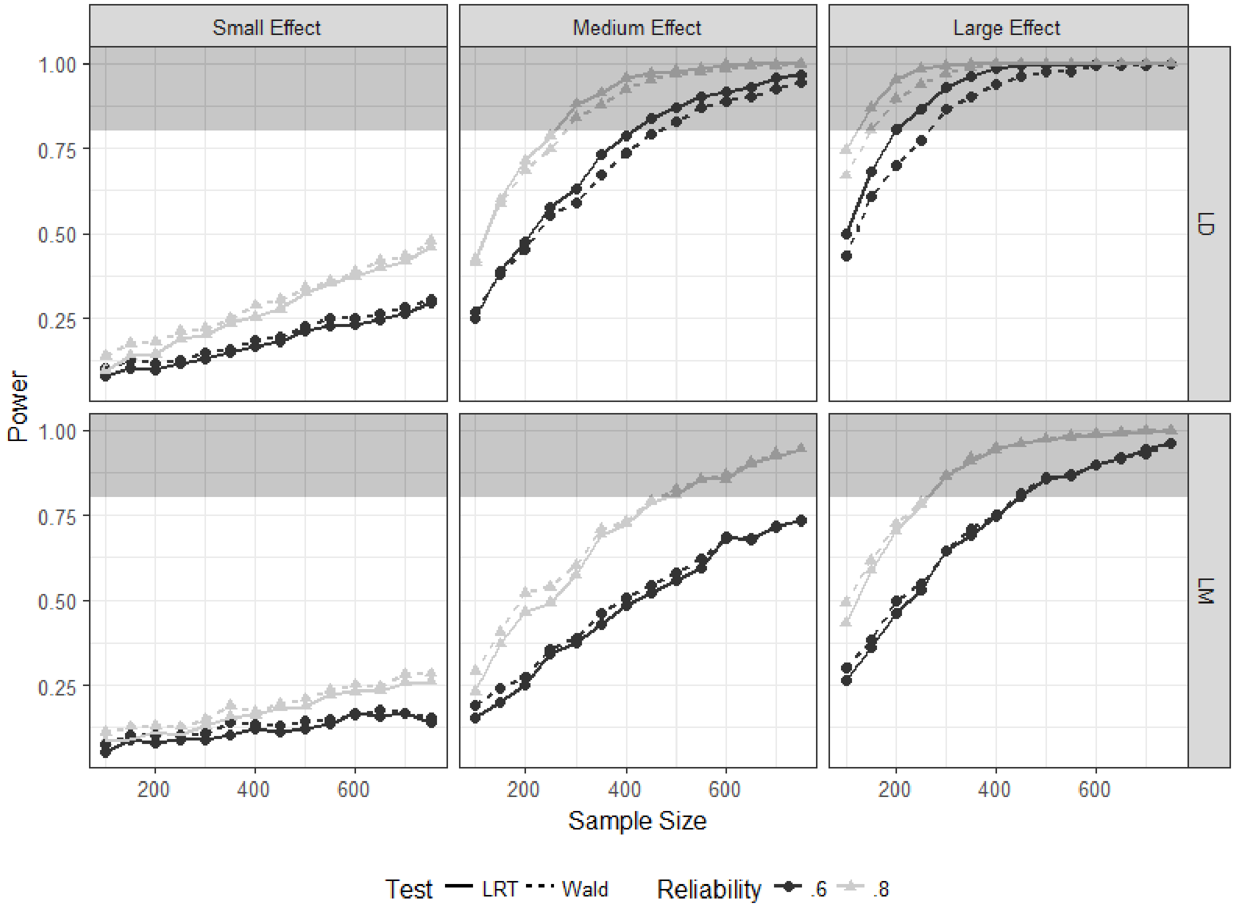

Figure A1. Power to detect quadratic trait-method correlations with the LD and LM models in simulated data sets. LRT=Likelihood ratio test; Wald=Wald test; LD=Latent Difference Model; LM=Latent Means model. Power of .8 and above is shaded.

MplusAutomation template code for the LD model simulation

```
#Set values for iterators to replace the appropriate values in the population model and sample size
[[init]]
iterators=ssize reliabilities effsize;
ssize=1:14;
reliabilities= 1 2 ;
effsize= 1 2 3;
szs#ssize= "100" "150" "200" "250" "300" "350" "400" "450" "500" "550" "600" "650" "700" "750";
relt#reliabilities="8" "6";
efft#effsize="smef" "medef" "lgef";
effv#effsize= "0.04" "0.103" "0.145";
filename="LD_m-d_in_t1_MC_7-20_Rel[[relt#reliabilities]]s[[szs#ssize]][[efft#effsize]].inp";
outputDirectory="C:/Users/Fred/Box Sync/Research/Nonlinear MTMM/Monte Carlo/Quadratic Diff Score/10-9-
LD_sim/Inputs";
[[/init]]

#Body of Mplus code, with values of iterators in brackets

Title: Monte Carlo procedure for testing quadratic difference score
estimation with parameter values saved from Burns Child behavior checklist
 data example.
Reliability of indicators is [[relt#reliabilities]]
Sample size is [[szs#ssize]]
effect size is [[efft#effsize]];

#Create variable names, assign locations for results and replications to be saved for all replications of the data
Montecarlo: names are IN1MomT1    IN2MomT1    IN3MomT1
    IN1dadT1     IN2dadT1     IN3dadT1;
            Nobservations =[[szs#ssize]];
            Nreps= 1000;
            Seed=84341;
results=LD_MC_10-9_Rel[[relt#reliabilities]]s[[szs#ssize]][[efft#effsize]]_results.sav;
repsave=ALL;
save=datfiles/s[[szs#ssize]]rel[[relt#reliabilities]][[efft#effsize]]/LD_MC_10-
9_s[[szs#ssize]]rel[[relt#reliabilities]][[efft#effsize]]_rep*.dat;

#Specify the population model
Model population:
    ! TMU factors IN
  ! Mother report IN
  INMOM by IN1momT1@1
  IN2momT1*1.201
    IN3momT1*1.075;
  ! dad report IN
  INdad by IN1dadT1@1
    IN2dadT1*1.201
      IN3dadT1*1.075;
  ! Mean structure and intercepts
  ! Set intercepts of reference indicators to zero
  [IN1momT1@0 IN1dadT1@0];
  ! Set remaining intercepts equal across raters
  [IN2momT1*.048 IN2dadT1*.048];
  [IN3momT1*.082 IN3dadT1*.082];
```

```
!add error variances
[[reliabilities = 2]]
 in1momt1*0.491 in2momt1*0.709 in3momt1*0.568 ;
 [[/reliabilities = 2]]
[[reliabilities = 1]]
 in1momt1*0.184 in2momt1*0.266 in3momt1*0.213 ;
 [[/reliabilities = 1]]

[[effsize=1]]
 [[reliabilities=1]]
 IN1dadt1*0.227 In2dadt1*0.343 In3dadt1*0.272; ! Eff is 0.0196 Rel is 0.8
 [[/reliabilities=1]]
  [[/effsize=1]]
 [[effsize=2]]
 [[reliabilities=1]]
 IN1dadt1*0.236 In2dadt1*0.356 In3dadt1*0.282; ! Eff is 0.1304 Rel is 0.8
 [[/reliabilities=1]]
  [[/effsize=2]]
 [[effsize=3]]
 [[reliabilities=1]]
 IN1dadt1*0.247 In2dadt1*0.372 In3dadt1*0.295; ! Eff is 0.2592 Rel is 0.8
 [[/reliabilities=1]]
  [[/effsize=3]]
 [[effsize=1]]
 [[reliabilities=2]]
 IN1dadt1*0.606 In2dadt1*0.915 In3dadt1*0.724; ! Eff is 0.0196 Rel is 0.6
 [[/reliabilities=2]]
  [[/effsize=1]]
 [[effsize=2]]
 [[reliabilities=2]]
 IN1dadt1*0.63 In2dadt1*0.95 In3dadt1*0.753; ! Eff is 0.1304 Rel is 0.6
 [[/reliabilities=2]]
  [[/effsize=2]]
 [[effsize=3]]
 [[reliabilities=2]]
 IN1dadt1*0.658 In2dadt1*0.992 In3dadt1*0.786; ! Eff is 0.2592 Rel is 0.6
 [[/reliabilities=2]]
  [[/effsize=3]]

  !Create diff variable
  INdaddif by IN1momT1@0;
  INdad on INMOM@1 INdadDIF@1;
  INdad@0;
  !INdadDIF with parcel2@0 parcel3@0;
  !Create quadratic term
   INMOMsq | INMOM xwith INMOM;
     INdaddif on INMOM*-.331 INMOMsq*[[effv#effsize]];
    ! Estimate latent means
  [INMOM*.955 INdadDIF*.224];


INdaddif*.224;

 INmom*.763;
```

```
#Specify analysis model
Model:    ! TMU factors IN
  ! Mother report IN
  INMOM by IN1momT1@1
  IN2momT1*1.228 (lambda2)
    IN3momT1*1.093 (lambda3);
  ! dad report IN
  INdad by IN1dadT1@1
    IN2dadT1*1.228 (lambda2)
      IN3dadT1*1.093 (lambda3);
  ! Mean structure and intercepts
  ! Set intercepts of reference indicators to zero
  [IN1momT1@0 IN1dadT1@0];
  ! Set remaining intercepts equal across raters
  [IN2momT1*.048 IN2dadT1*.048] (alpha2);
  [IN3momT1*.082 IN3dadT1*.082] (alpha3);

[[reliabilities = 2]]
 in1momt1*0.491 in2momt1*0.709 in3momt1*0.568 ;
 [[/reliabilities = 2]]

[[reliabilities = 1]]
 in1momt1*0.184 in2momt1*0.266 in3momt1*0.213 ;
 [[/reliabilities = 1]]


[[effsize=1]]
 [[reliabilities=1]]
 IN1dadt1*0.227 In2dadt1*0.343 In3dadt1*0.272; ! Eff is 0.0196 Rel is 0.8
 [[/reliabilities=1]]
  [[/effsize=1]]
 [[effsize=2]]
 [[reliabilities=1]]
 IN1dadt1*0.236 In2dadt1*0.356 In3dadt1*0.282; ! Eff is 0.1304 Rel is 0.8
 [[/reliabilities=1]]
  [[/effsize=2]]
 [[effsize=3]]
 [[reliabilities=1]]
 IN1dadt1*0.247 In2dadt1*0.372 In3dadt1*0.295; ! Eff is 0.2592 Rel is 0.8
 [[/reliabilities=1]]
  [[/effsize=3]]
 [[effsize=1]]
 [[reliabilities=2]]
 IN1dadt1*0.606 In2dadt1*0.915 In3dadt1*0.724; ! Eff is 0.0196 Rel is 0.6
 [[/reliabilities=2]]
  [[/effsize=1]]
 [[effsize=2]]
 [[reliabilities=2]]
 IN1dadt1*0.63 In2dadt1*0.95 In3dadt1*0.753; ! Eff is 0.1304 Rel is 0.6
 [[/reliabilities=2]]
  [[/effsize=2]]
 [[effsize=3]]
 [[reliabilities=2]]
 IN1dadt1*0.658 In2dadt1*0.992 In3dadt1*0.786; ! Eff is 0.2592 Rel is 0.6
 [[/reliabilities=2]]
  [[/effsize=3]]
```

```
  !Create diff variable
  INdaddif by IN1momT1@0;
  INdad on INMOM@1 INdadDIF@1;
  INdad@0;
  !INdadDIF with parcel2@0 parcel3@0;
  !Create quadratic term
   INMOMsq | INMOM xwith INMOM;
     INdaddif on INMOM*-.331 INMOMsq*[[effv#effsize]];
    ! Estimate latent means
  [INMOM*.955 INdadDIF*.224];


INdaddif*.224;

 INmom*.763;
 # Set analysis settings to select LMS, and increase iterations and quadrature points.
analysis: type=random; algorithm=integration;
integration=GAUSSHERMITE (50);
 adaptive=OFF;
 stiterations=50;
 iterations=5000;
  sditerations=250;
  miterations=1000;

#Request tech9 output
output: tech9;
```

MplusAutomation Template Code for the LM Model simulation for Type 1 error

```
#Set values for iterators to replace the appropriate values in the population model and sample size
[[init]]
iterators=ssize rel conv;
ssize=1 2 3 4 5 6 7 8 9 10 11 12 13 14;
rel=1 2 3 4;
conv=1 2 3 4 5;
reltext#rel = "rel9" "rel8" "rel7" "rel6";
convtext#conv= "rsq005" "rsq01" "rsq03" "rsq05" "rsq07";
szs#ssize= "100" "150" "200" "250" "300" "350" "400" "450" "500" "550" "600" "650" "700" "750";
filename="MC_MECOM_linear_mtht1vals_[[reltext#rel]]_s[[szs#ssize]]_conv[[convtext#conv]]_savedreps.inp";
outputDirectory="C:/Users/Fred/Box Sync/Research/Nonlinear MTMM/Monte Carlo/Quadratic Diff Score/8-7-
17_create_difftest_function/Inputs/datfiles";
[[/init]]

#Body of Mplus code, with values of iterators in brackets

title: LM Monte Carlo cell Sample size= [[szs#ssize]]
r squared of latent regression= [[convtext#conv]];
reliability = [[reltext#rel]];

#Tells mplus to read previously generated data from other monte carlo runs
data:
file=s[[szs#ssize]][[reltext#rel]][[convtext#conv]]/MC_MECOMq_mtht1valsq_[[reltext#rel]]_s[[szs#ssize]]_conv[[
convtext#conv]]replist.dat;

type=MONTECARLO;
 ANALYSIS: estimator=ML; process=8;
  variable:
      names are him1 him2 him3 hit1 hit2 hit3;
       usevariables =him1 him2 him3 hit1 hit2 hit3;

#Model is specified as a linear LM model (Pohl et al., 2010)
Model:
! TMU factors HI
  ! Common HI Factor
  HICOM by HIm1@1
                                HIm2*.983 (lambda2b)
                                 HIm3*.963 (lambda3b)
    HIt1@1
    HIt2*.983 (lambda2b)
      HIt3*.963 (lambda3b);

  !Create method variable

  Meth by him1@-1
  him2*-.983 (lambda2a)
    him3*-.963 (lambda3a)
    HIt1@1
    HIt2*.983 (lambda2b)
      HIt3*.963 (lambda3b);


!Regression Values
    Meth on HICOM*1.73;
```

```
!latent means
  [HICOM*1.024 Meth*1.47];

!Intercepts
[him1@0 hit1@0];

  ! Set remaining intercepts equal across raters
  [him2*-.118 hit2*-.118](alpha2);
  [him3*.024 hit3*.024] (alpha3);


!Variance
Hicom*0.906;

!Residual Variance of Method Factor
[[conv=1]]
Meth*3.56;
[[/conv=1]]

[[conv=2]]
Meth*1.686;
[[/conv=2]]

[[conv=3]]
Meth*0.437;
[[/conv=3]]

[[conv=4]]
Meth*0.187;
[[/conv=4]]

[[conv=5]]
Meth*0.08;
[[/conv=5]]

! Different residual variances
[[rel = 4]]
 [[conv = 1]]
 him1*3.181 him2*3.073 him3*2.95 ;
 [[/conv = 1]]
 [[/rel = 4]]

[[rel = 3]]
 [[conv = 1]]
 him1*2.045 him2*1.976 him3*1.896 ;
 [[/conv = 1]]
 [[/rel = 3]]

[[rel = 2]]
 [[conv = 1]]
 him1*1.193 him2*1.153 him3*1.106 ;
 [[/conv = 1]]
 [[/rel = 2]]

[[rel = 1]]
```

```
 [[conv = 1]]
 him1*0.53 him2*0.512 him3*0.492 ;
 [[/conv = 1]]
 [[/rel = 1]]

[[rel = 4]]
 [[conv = 1]]
 hit1*3.181 hit2*3.073 hit3*2.95 ;
 [[/conv = 1]]
 [[/rel = 4]]

[[rel = 3]]
 [[conv = 1]]
 hit1*2.045 hit2*1.976 hit3*1.896 ;
 [[/conv = 1]]
 [[/rel = 3]]

[[rel = 2]]
 [[conv = 1]]
 hit1*1.193 hit2*1.153 hit3*1.106 ;
 [[/conv = 1]]
 [[/rel = 2]]

[[rel = 1]]
 [[conv = 1]]
 hit1*0.53 hit2*0.512 hit3*0.492 ;
 [[/conv = 1]]
 [[/rel = 1]]

[[rel = 4]]
 [[conv = 2]]
 him1*1.932 him2*1.867 him3*1.791 ;
 [[/conv = 2]]
 [[/rel = 4]]

[[rel = 3]]
 [[conv = 2]]
 him1*1.242 him2*1.2 him3*1.152 ;
 [[/conv = 2]]
 [[/rel = 3]]

[[rel = 2]]
 [[conv = 2]]
 him1*0.724 him2*0.7 him3*0.672 ;
 [[/conv = 2]]
 [[/rel = 2]]

[[rel = 1]]
 [[conv = 2]]
 him1*0.322 him2*0.311 him3*0.299 ;
 [[/conv = 2]]
 [[/rel = 1]]

[[rel = 4]]
 [[conv = 2]]
 hit1*1.932 hit2*1.867 hit3*1.791 ;
```

```
 [[/conv = 2]]
 [[/rel = 4]]

[[rel = 3]]
 [[conv = 2]]
 hit1*1.242 hit2*1.2 hit3*1.152 ;
 [[/conv = 2]]
 [[/rel = 3]]

[[rel = 2]]
 [[conv = 2]]
 hit1*0.724 hit2*0.7 hit3*0.672 ;
 [[/conv = 2]]
 [[/rel = 2]]

[[rel = 1]]
 [[conv = 2]]
 hit1*0.322 hit2*0.311 hit3*0.299 ;
 [[/conv = 2]]
 [[/rel = 1]]

[[rel = 4]]
 [[conv = 3]]
 him1*1.099 him2*1.062 him3*1.019 ;
 [[/conv = 3]]
 [[/rel = 4]]

[[rel = 3]]
 [[conv = 3]]
 him1*0.706 him2*0.683 him3*0.655 ;
 [[/conv = 3]]
 [[/rel = 3]]

[[rel = 2]]
 [[conv = 3]]
 him1*0.412 him2*0.398 him3*0.382 ;
 [[/conv = 3]]
 [[/rel = 2]]

[[rel = 1]]
 [[conv = 3]]
 him1*0.183 him2*0.177 him3*0.17 ;
 [[/conv = 3]]
 [[/rel = 1]]

[[rel = 4]]
 [[conv = 3]]
 hit1*1.099 hit2*1.062 hit3*1.019 ;
 [[/conv = 3]]
 [[/rel = 4]]
[[rel = 3]]
 [[conv = 3]]
 hit1*0.706 hit2*0.683 hit3*0.655 ;
 [[/conv = 3]]
 [[/rel = 3]]
[[rel = 2]]
```

```
 [[conv = 3]]
 hit1*0.412 hit2*0.398 hit3*0.382 ;
 [[/conv = 3]]
 [[/rel = 2]]
[[rel = 1]]
 [[conv = 3]]
 hit1*0.183 hit2*0.177 hit3*0.17 ;
 [[/conv = 3]]
 [[/rel = 1]]
[[rel = 4]]
 [[conv = 4]]
 him1*0.932 him2*0.901 him3*0.865 ;
 [[/conv = 4]]
 [[/rel = 4]]
[[rel = 3]]
 [[conv = 4]]
 him1*0.599 him2*0.579 him3*0.556 ;
 [[/conv = 4]]
 [[/rel = 3]]
[[rel = 2]]
 [[conv = 4]]
 him1*0.35 him2*0.338 him3*0.324 ;
 [[/conv = 4]]
 [[/rel = 2]]
[[rel = 1]]
 [[conv = 4]]
 him1*0.155 him2*0.15 him3*0.144 ;
 [[/conv = 4]]
 [[/rel = 1]]
[[rel = 4]]
 [[conv = 4]]
 hit1*0.932 hit2*0.901 hit3*0.865 ;
 [[/conv = 4]]
 [[/rel = 4]]
[[rel = 3]]
 [[conv = 4]]
 hit1*0.599 hit2*0.579 hit3*0.556 ;
 [[/conv = 4]]
 [[/rel = 3]]
[[rel = 2]]
 [[conv = 4]]
 hit1*0.35 hit2*0.338 hit3*0.324 ;
 [[/conv = 4]]
 [[/rel = 2]]
[[rel = 1]]
 [[conv = 4]]
 hit1*0.155 hit2*0.15 hit3*0.144 ;
 [[/conv = 4]]
 [[/rel = 1]]
[[rel = 4]]
 [[conv = 5]]
 him1*0.861 him2*0.832 him3*0.799 ;
 [[/conv = 5]]
 [[/rel = 4]]
[[rel = 3]]
 [[conv = 5]]
```

```
 him1*0.554 him2*0.535 him3*0.513 ;
 [[/conv = 5]]
 [[/rel = 3]]
[[rel = 2]]
 [[conv = 5]]
 him1*0.323 him2*0.312 him3*0.299 ;
 [[/conv = 5]]
 [[/rel = 2]]
[[rel = 1]]
 [[conv = 5]]
 him1*0.144 him2*0.139 him3*0.133 ;
 [[/conv = 5]]
 [[/rel = 1]]
[[rel = 4]]
 [[conv = 5]]
 hit1*0.861 hit2*0.832 hit3*0.799 ;
 [[/conv = 5]]
 [[/rel = 4]]
[[rel = 3]]
 [[conv = 5]]
 hit1*0.554 hit2*0.535 hit3*0.513 ;
 [[/conv = 5]]
 [[/rel = 3]]
[[rel = 2]]
 [[conv = 5]]
 hit1*0.323 hit2*0.312 hit3*0.299 ;
 [[/conv = 5]]
 [[/rel = 2]]
[[rel = 1]]
 [[conv = 5]]
 hit1*0.144 hit2*0.139 hit3*0.133 ;
 [[/conv = 5]]
 [[/rel = 1]]

  model constraint:
  0 = lambda2b + lambda2a;
  0 = lambda3b + lambda3a;


output: tech1 tech9;

savedata: results=MC_MECOM_linear_mtht1vals_[[reltext#rel]]_s[[szs#ssize]]_conv[[convtext#conv]]_results.sav;
```

Appendix C

Mplus Code for Specifying the LM and LD Models

LD Model with no quadratic effect

```
Title:
  Wave 1, Inattention mother vs contrastnames#contrast model

Data:
  File is cgINHIAIt1t2t3.dat ;

Variable:
  Names are   IN1momT1    IN2momT1    IN3momT1
  IN1dadT1    IN2dadT1    IN3dadT1;

Usevariables are

IN1MomT1    IN2MomT1    IN3MomT1
  IN1dadT1     IN2dadT1     IN3dadT1;

Missing are . ;

ANALYSIS: estimator=ML

Model:

  ! TMU factors IN
! Mother report IN
INMOM by IN1momT1@1
IN2momT1 (lambda2)
  IN3momT1 (lambda3);

! dad report IN
INdad by IN1dadT1@1
  IN2dadT1 (lambda2)
    IN3dadT1 (lambda3);

! Mean structure and intercepts
! Set intercepts of reference indicators to zero
[IN1momT1@0 IN1dadT1@0];

! Set remaining intercepts equal across raters
```

```
[IN2momT1 IN2dadT1](alpha2);
[IN3momT1 IN3dadT1] (alpha3);

!Correlated Errors of identical parcels
IN2momT1 with IN2dadT1;
IN3momT1 with IN3dadT1;

!Create diff variable

INdaddif by IN1momT1@0;
INdad on INMOM@1 INdadDIF@1;
INdad@0;

  ! Estimate latent means
[INMOM* INdadDIF*];
Output:  sampstat;
plot: type=plot3;
```

LD Model with a quadratic effect

```
Title:
  Wave 1, Inattention mother vs contrastnames#contrast model

Data:
  File is cgINHIAIt1t2t3.dat ;

Variable:
  Names are   IN1momT1    IN2momT1    IN3momT1
  IN1dadT1    IN2dadT1    IN3dadT1;

Usevariables are

IN1MomT1    IN2MomT1    IN3MomT1
  IN1dadT1     IN2dadT1     IN3dadT1;

Missing are . ;

ANALYSIS: type=random; algorithm=integration; integration=GAUSSHERMITE (50);
     adaptive=OFF; stiterations=50; iterations=5000; sditerations=250;
     miterations=1000; process=8;

Model:

  ! TMU factors IN
! Mother report IN
INMOM by IN1momT1@1
IN2momT1 (lambda2)
  IN3momT1 (lambda3);


! dad report IN
INdad by IN1dadT1@1
  IN2dadT1 (lambda2)
    IN3dadT1 (lambda3);

! Mean structure and intercepts
! Set intercepts of reference indicators to zero
[IN1momT1@0 IN1dadT1@0];

! Set remaining intercepts equal across raters
[IN2momT1 IN2dadT1](alpha2);
[IN3momT1 IN3dadT1] (alpha3);
```

```
! P - 1 Parcel-specific method factors

!Parcel2 by IN2momT1 IN2dadT1;
!Parcel3 by IN3momT1 IN3dadT1;

!INMOM INdad with Parcel2@0 Parcel3@0;

IN2momT1 with IN2dadT1;
IN3momT1 with IN3dadT1;

!Create diff variable

INdaddif by IN1momT1@0;
INdad on INMOM@1 INdadDIF@1;
INdad@0;

!INdadDIF with parcel2@0 parcel3@0;

!Create quadratic term
 INMOMsq | INMOM xwith INMOM;


   INdaddif on INMOM INMOMsq;

  ! Estimate latent means
[INMOM* INdadDIF*];

Output:  sampstat;
plot: type=plot3;
```

LM Model with no quadratic effect

```
Title:
  Wave 1, Inattention mother vs contrastnames#contrast model

Data:
  File is cgINHIAIt1t2t3.dat ;

Variable:
  Names are   IN1momT1    IN2momT1    IN3momT1
  IN1dadT1    IN2dadT1    IN3dadT1;

Usevariables are

IN1MomT1    IN2MomT1    IN3MomT1
  IN1dadT1     IN2dadT1     IN3dadT1;

Missing are . ;

ANALYSIS: estimator=ML

Model:
  ! TMU factors IN
! Common IN Factor
INCOM by IN1momT1@1
IN2momT1 (lambda2a)
  IN3momT1 (lambda3a)
  IN1dadT1@1
  IN2dadT1 (lambda2a)
    IN3dadT1 (lambda3a);

! Mean structure and intercepts
! Set intercepts of reference indicators to zero
[IN1momT1@0 IN1dadT1@0];

! Set remaining intercepts equal across raters
[IN2momT1 IN2dadT1](alpha2);
[IN3momT1 IN3dadT1] (alpha3);

!Correlated errors for identical parcels
IN2momT1 with IN2dadT1;
IN3momT1 with IN3dadT1;

!Create method variable

Meth by IN1momT1@-1
```

```
IN2momT1*-1(lambda2b)
  IN3momT1*-1(lambda3b)
  IN1dadT1@1
  IN2dadT1*1(lambda2a)
    IN3dadT1*1 (lambda3a);

  Meth on INCOM INCOMsq;

  ! Estimate latent means
[INCOM* Meth*];

Output:  sampstat;
plot: type=plot3;
```

Mplus code for LM model with quadratic effect included

```
Title:
  Wave 1, Inattention mother vs contrastnames#contrast model

Data:
  File is cgINHIAIt1t2t3.dat ;

Variable:
  Names are   IN1momT1    IN2momT1    IN3momT1
  IN1dadT1    IN2dadT1    IN3dadT1;

Usevariables are

IN1MomT1    IN2MomT1    IN3MomT1
  IN1dadT1     IN2dadT1     IN3dadT1;

Missing are . ;

ANALYSIS: type=random; algorithm=integration; integration=GAUSSHERMITE (50);
     adaptive=OFF; stiterations=50; iterations=5000; sditerations=250;
     miterations=1000; processors=4

Model:

  ! TMU factors IN
! Common IN Factor
INCOM by IN1momT1@1
IN2momT1 (lambda2a)
  IN3momT1 (lambda3a)
  IN1dadT1@1
```

```
  IN2dadT1 (lambda2a)
    IN3dadT1 (lambda3a);

! Mean structure and intercepts
! Set intercepts of reference indicators to zero
[IN1momT1@0 IN1dadT1@0];

! Set remaining intercepts equal across raters
[IN2momT1 IN2dadT1](alpha2);
[IN3momT1 IN3dadT1] (alpha3);

!Correlated errors for identical parcels
IN2momT1 with IN2dadT1;
IN3momT1 with IN3dadT1;

!Create method variable

Meth by IN1momT1@-1
IN2momT1*-1(lambda2b)
  IN3momT1*-1(lambda3b)
  IN1dadT1@1
  IN2dadT1*1(lambda2a)
    IN3dadT1*1 (lambda3a);

!INdadDIF with parcel2@0 parcel3@0;

!Create quadratic term
 INCOMsq | INCOM xwith INCOM;

  Meth on INCOM INCOMsq;

  ! Estimate latent means
[INCOM* Meth*];

Output:  sampstat;
plot: type=plot3;
```
